# GWAS for serum galactose-deficient IgA1 implicates critical genes of the *O*-glycosylation pathway

**DOI:** 10.1371/journal.pgen.1006609

**Published:** 2017-02-10

**Authors:** Krzysztof Kiryluk, Yifu Li, Zina Moldoveanu, Hitoshi Suzuki, Colin Reily, Ping Hou, Jingyuan Xie, Nikol Mladkova, Sindhuri Prakash, Clara Fischman, Samantha Shapiro, Robert A. LeDesma, Drew Bradbury, Iuliana Ionita-Laza, Frank Eitner, Thomas Rauen, Nicolas Maillard, Francois Berthoux, Jürgen Floege, Nan Chen, Hong Zhang, Francesco Scolari, Robert J. Wyatt, Bruce A. Julian, Ali G. Gharavi, Jan Novak

**Affiliations:** 1 Dept. of Medicine, Div. of Nephrology, College of Physicians and Surgeons, Columbia University, New York, New York, United States of America; 2 Dept. of Microbiology, University of Alabama at Birmingham, Birmingham, Alabama, United States of America; 3 Division of Nephrology, Dept. of Internal Medicine, Juntendo University Faculty of Medicine, Tokyo, Japan; 4 Dept. of Medicine, University of Alabama at Birmingham, Birmingham, Alabama, United States of America; 5 Renal Div., Peking University First Hospital, Peking University Institute of Nephrology, Beijing, China; 6 Dept. of Nephrology, Ruijin Hospital, Shanghai Jiao Tong University School of Medicine, Shanghai, China; 7 Dept. of Biostatistics, Mailman School of Public Health, Columbia University, New York, New York, United States of America; 8 Dept. of Nephrology, RWTH University of Aachen, Aachen, Germany; 9 Kidney Diseases Research, Bayer Pharma AG, Wuppertal, Germany; 10 Nephrology, Dialysis, and Renal Transplantation Dept., University North Hospital, Saint Etienne, France; 11 Div. of Nephrology, Azienda Ospedaliera Spedali Civili of Brescia, Montichiari Hospital, Univ of Brescia, Brescia, Italy; 12 Dept. of Medical and Surgical Specialties, Radiological Sciences, University of Brescia, Brescia, Italy; 13 Div. of Pediatric Nephrology, University of Tennessee Health Sciences Center, Memphis, Tennessee, United States of America; 14 Children's Foundation Research Institute, Le Bonheur Children's Hospital, Memphis, Tennessee, United States of America; University of Miami, Miller School of Medicine, UNITED STATES

## Abstract

Aberrant *O*-glycosylation of serum immunoglobulin A1 (IgA1) represents a heritable pathogenic defect in IgA nephropathy, the most common form of glomerulonephritis worldwide, but specific genetic factors involved in its determination are not known. We performed a quantitative GWAS for serum levels of galactose-deficient IgA1 (Gd-IgA1) in 2,633 subjects of European and East Asian ancestry and discovered two genome-wide significant loci, in *C1GALT1* (rs13226913, *P* = 3.2 x 10^−11^) and *C1GALT1C1* (rs5910940, *P* = 2.7 x 10^−8^). These genes encode molecular partners essential for enzymatic *O*-glycosylation of IgA1. We demonstrated that these two loci explain approximately 7% of variability in circulating Gd-IgA1 in Europeans, but only 2% in East Asians. Notably, the Gd-IgA1-increasing allele of rs13226913 is common in Europeans, but rare in East Asians. Moreover, rs13226913 represents a strong cis-eQTL for *C1GALT1* that encodes the key enzyme responsible for the transfer of galactose to *O*-linked glycans on IgA1. By *in vitro* siRNA knock-down studies, we confirmed that mRNA levels of both *C1GALT1* and *C1GALT1C1* determine the rate of secretion of Gd-IgA1 in IgA1-producing cells. Our findings provide novel insights into the genetic regulation of *O*-glycosylation and are relevant not only to IgA nephropathy, but also to other complex traits associated with *O*-glycosylation defects, including inflammatory bowel disease, hematologic disease, and cancer.

## Author summary

*O*-glycosylation is a common type of post-translational modification of proteins; specific abnormalities in the mechanism of *O*-glycosylation have been implicated in cancer, inflammatory and blood diseases. However, the molecular basis of abnormal *O*-glycosylation in these complex disorders is not known. We studied the genetic basis of defective *O*-glycosylation of serum immunoglobulin A1 (IgA1), that represents the key pathogenic defect in IgA nephropathy, the most common form of primary glomerulonephritis worldwide. We report our results of the first genome-wide association study for this trait using serum assays in 2,633 individuals of European and East-Asian ancestry. In our genome scan, we observed two significant signals with large effects, on chromosomes 7p21.3 and Xq24, jointly explaining about 7% of trait variability. These signals implicate two genes that encode molecular partners essential for enzymatic *O*-glycosylation of IgA1 and mucins, and represent potential new targets for therapy.

## Introduction

*N*- and *O*-glycosylation are fundamental post-translational modifications of proteins in mammalian cells. Abnormalities in glycosylation have been linked to a broad range of human diseases, including neurologic disorders, immune-mediated and inflammatory diseases as well as cancer. Protein glycosylation is mediated by a large family of enzymes that have cell- and tissue-specific activity, and can generate highly diverse glycan structures that are important for signaling, cell-cell and cell-matrix interactions. The combinatorial possibilities of glycan structures imparted by the large number of glycosylation enzymes complicate a systematic analysis of protein glycosylation patterns and identification of critical steps involved in the activity, concentration, and regulation in any given cell or tissue. In such a setting, genetic studies of congenital defects of glycosylation in humans have provided significant insight into non-redundant regulatory nodes in this pathway[1]. The majority of these Mendelian disorders arise from loss of function mutations that severely perturb protein glycosylation across a range of tissues and produce a wide range of organ dysfunction in early life. However, less pronounced abnormalities in protein glycosylation have also been detected in complex disorders such as autoimmunity and cancer, suggesting that more subtle defects in this pathway can have important consequences for human health.

IgA nephropathy (IgAN), the most common cause of glomerulonephritis and a common cause of kidney failure worldwide, is a prototypical example of an immune-mediated disorder characterized by abnormal glycosylation[2]. In humans, the hinge-region segments of the heavy chains of immunoglobulin A1 (IgA1) have 3 to 6 *O*-glycans, resulting in a variety of IgA1 glycoforms in circulation. In healthy individuals, the prevailing *O*-glycans include the *N*-acetylgalactosamine (GalNAc)-galactose disaccharide and its sialylated forms. In IgAN, galactose-deficient IgA1 (Gd-IgA1) glycoforms are significantly more abundant compared to those of healthy controls[3]. These under-galactosylated glycoforms are secreted by IgA1-producing cells while galactosylation of other circulating *O*-glycosylated proteins is preserved, suggesting a specific defect within IgA1-producing cells[4]. The pathogenetic mechanism of IgAN involves an autoimmune response resulting in production of IgA or IgG autoantibodies against circulating Gd-IgA1, and formation of immune complexes (Gd-IgA1 complexed with autoantibodies) that deposit in the kidney and cause tissue injury[2, 5]. Consistent with this mechanism, Gd-IgA1 is the predominant glycoform in circulating immune complexes and in the glomerular immune deposits in patients with IgAN[6–9] and elevated serum levels of Gd-IgA1 (autoantigen) and anti-glycan antibodies (autoantibody) are associated with more aggressive disease and accelerated progression to end-stage kidney failure[10, 11].

The design of a simple lectin-based ELISA assay, using a GalNAc-specific lectin from *Helix aspersa* (HAA), enables screening of sera to quantify the levels of circulating Gd-IgA1[3]. Using this assay, we have demonstrated that the serum levels of Gd-IgA1 represent a normally distributed quantitative trait in healthy populations, but up to two thirds of IgAN patients have levels above the 95^th^ percentile for healthy controls. Examining family members of probands with familial and sporadic forms of IgAN, we also showed that elevated serum Gd-IgA1 levels segregate independently of serum total IgA levels and have high heritability (estimated at 50–70%)[12, 13]. Moreover, many healthy family members exhibited very high Gd-IgA1 levels, identifying elevated Gd-IgA1 as a heritable risk factor that precedes the development of IgAN.

To date, multiethnic genome-wide association studies involving over 20,000 individuals have identified 15 risk loci predisposing to IgAN, highlighting the importance of innate and adaptive immunity in this disorder. Power analyses indicated that discovery of additional risk loci using the case-control design will require significant expansion in sample size. However, a systematic analysis of quantitative endophenotypes that are linked to disease pathogenesis, such as Gd-IgA1, has not been conducted to date and may provide the opportunity to discover additional pathogenic pathways using a smaller sample size. In this study, we performed the first GWAS for serum Gd-IgA1 levels, and successfully mapped new loci with surprisingly large contributions to the heritability of the circulating level of Gd-IgA1 independently of IgA levels.

## Results

**Fig 1:**
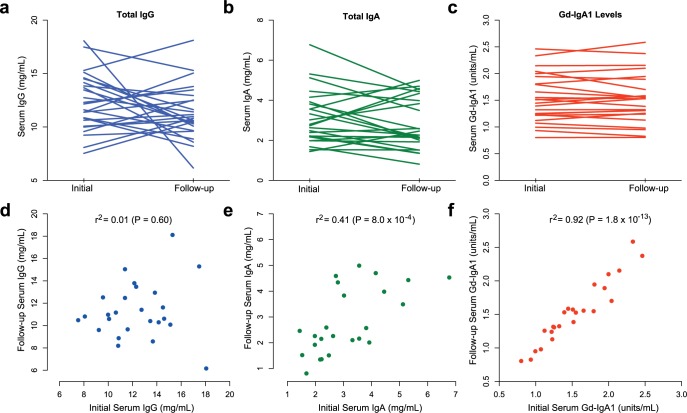
Longitudinal measurements of serum immunoglobulin levels and Gd-IgA1 levels over 4 years of follow-up. Initial and 4-year follow-up levels of **(a)** serum total IgG, **(b)** serum total IgA, and **(c)** serum Gd-IgA1 normalized for serum total IgA. Panels **(d, e, f)** represent scatter plots of initial (x-axis) versus follow-up (y-axis) values. P-values correspond to the Pearson’s test of correlation; r^2^: squared correlation coefficient.

In order to test if serum levels of Gd-IgA1 remain stable over time, we first performed measurements of total serum immunoglobulin levels along with Gd-IgA1 levels at baseline and at four years of follow-up in 32 individuals of European ancestry followed longitudinally (**Fig 1**). While serum total IgG and IgA levels varied with time, Gd-IgA1 levels (normalized for total IgA) remained remarkably stable over a 4-year period (r^2^ = 0.92, P = 1.8 x 10^−13^), demonstrating that *O*-glycosylation of IgA1 is minimally affected by random environmental factors.

We next used HAA lectin-based ELISA to analyze single time-point sera of 1,195 individuals in our discovery cohorts composed of 950 individuals of East-Asian ancestry (483 biopsy-documented IgAN cases and 467 controls) and 245 individuals of European ancestry (141 biopsy-documented IgAN cases and 104 controls, **Table 1**). As previously reported, serum Gd-IgA1 levels were positively correlated with age (East Asians r = 0.13, P = 8.9x10^-5^; Europeans r = 0.15, P = 1.7x10^-2^) and total IgA levels (East Asians r = 0.75, P < 2.2x10^-16^; Europeans r = 0.56, P < 2.2x10^-16^), but were independent of gender (P > 0.05). In both cohorts, Gd-IgA1 levels were also significantly higher in IgAN cases compared to controls independently of age and total IgA levels (adjusted P < 2.2x10^-16^ in each individual cohort), providing a large-scale replication of prior findings.

**Table 1:** Study cohorts after implementation of all quality control filters: the final counts of cases and controls by cohort are provided.

GWAS Cohorts*	Ancestry	N_Cases_	N_Controls_	N_Total_	Genotyping Rate	Genotyping Platform**
Chinese Discovery Cohort	East Asian	483	467	950	99.9%	Illumina 660-quad
US Discovery Cohort	European	141	104	245	99.8%	Illumina 550v3
**Total Discovery:**		**624**	**571**	**1,195**		
Japanese Replication Cohort	East Asian	122	80	202	99.5%	KASP^TM^, LGC Genomics
Chinese Replication Cohort	East Asian	451	0	451	98.5%	KASP^TM^, LGC Genomics
German Replication Cohort	European	191	164	355	99.2%	KASP^TM^, LGC Genomics
French Replication Cohort	European	74	0	74	99.2%	KASP^TM^, LGC Genomics
US Replication Cohort	European	122	234	356	99.0%	KASP^TM^, LGC Genomics
**Total Replication:**		**960**	**478**	**1,438**		
**Total All Cohorts:**		**1,584**	**1,049**	**2,633**		

* Only individuals with the overall genotyping rate >95% (discovery) or >90% (replication) were included in the analysis.

** KASP^TM^: Kompetitive Alelle Specific PCR (a proprietary SNP-typing technology by LGC Genomics; accuracy >99.8%).

We next performed a GWAS for serum levels of Gd-IgA1 in these cohorts with and without adjustment for total IgA levels. For genome-wide analysis, we used a linear model with individual SNPs coded as additive genetic predictors, and the outcome defined as standardized residuals of serum Gd-IgA1 after normalization and additional adjustment for case/control status, age, ancestry and cohort membership (see Methods). Each ethnicity-defined discovery cohort was analyzed separately and the results were meta-analyzed to prioritize top signals for follow-up. With this approach, we observed minimal genomic inflation in the combined genome-wide analyses (*λ* = 1.01), indicating negligible effect of population stratification.

We first examined potential associations with known IgAN susceptibility loci, but found no statistically significant or suggestive signals between Gd-IgA1 levels and known IgAN risk alleles (**S1 Table)**. In addition, we found no association between the global polygenetic risk score for IgAN, which captures the combined effect of all IgAN risk loci, and Gd-IgA1 levels. We also did not detect any associations of Gd-IgA1 levels with loci previously linked to variation in total IgA levels[14–16], IgA deficiency[17], or *N*-glycosylation of IgG[18]. At the same time, we replicated previously reported association of total IgA with *ELL2* (rs56219066, P = 8.5x10^-3^)[15], confirming that genetic regulation of IgA levels is distinct from that for Gd-IgA1 levels. These data thus indicated the presence of yet undiscovered loci controlling variation in Gd-IgA1 levels.

We next examined genome-wide distribution of P-values from the discovery stage to identify novel loci associated with Gd-IgA1 levels. Although no signal reached genome-wide significance in the discovery stage, we observed several suggestive (P < 5x10^-4^) loci that we followed up in 1,438 additional individuals of East-Asian (N = 653) and European (N = 785) ancestry (**S1 Fig**). Subsequently, we analyzed all cohorts (N = 2,633) jointly to identify genome-wide significant loci (**Table 2, S2 Table**). Our power calculations demonstrate that our design provides adequate power to detect variants explaining ≥1.5% of overall trait variance at a genome-wide significant alpha 5x10^-8^ (**S3 Table**).

**Table 2:** Combined results for new significant and suggestive GWAS signals: serum Gd-IgA1 levels were determined using HAA lectin-based ELISA, normalized and adjusted for age, case-control status and serum total IgA levels.

				Discovery Cohorts			Replication Cohorts			All Cohorts				
				N = 1,195			N = 1,438			N = 2,633				
Chr	Position (kb)	SNP	Allele*	Effect	SE	P-value	Effect	SE	P-value	Effect	SE	P-value	Hetero** P-value	Genes in Locus
7	7213	rs13226913	**T**	0.20	0.05	2.2E-04	0.23	0.04	3.0E-08	0.22	0.03	3.2E-11	0.43 (NS)	***C1GALT1***
7	7239	rs1008897	**G**	0.19	0.06	4.6E-04	0.22	0.04	4.6E-07	0.21	0.03	9.1E-10	0.93 (NS)	***C1GALT1***
X	119642	rs5910940	**T**	0.13	0.03	2.3E-04	0.11	0.03	3.8E-05	0.14	0.03	2.7E-08	0.88 (NS)	***C1GALT1C1***
X	119698	rs2196262	**A**	0.11	0.03	1.2E-03	0.10	0.03	3.3E-04	0.12	0.02	1.4E-06	0.46 (NS)	***C1GALT1C1***
7	43345	rs978056	**G**	0.10	0.03	1.2E-03	0.07	0.03	7.5E-03	0.08	0.02	3.3E-05	0.15 (NS)	***HECW1***

* Gd-IgA1-increasing allele is provided as reference

** P-value for the test of heterogeneity of effects

**Fig 2:**
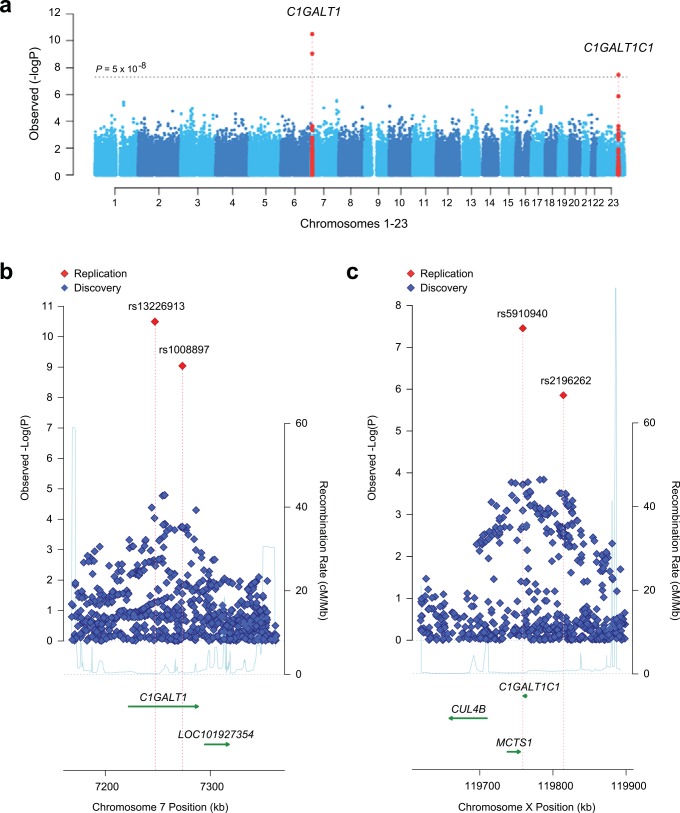
Combined meta-analysis of serum Gd-IgA1 levels in 2,633 individuals of European and East-Asian ancestry. Manhattan plot **(a)**, and regional plots for two distinct genome-wide significant loci: the *C1GALT1* locus **(b)** and the *C1GALT1C1* locus **(c).** The physical distance in kilobases (kb) is depicted along the x-axis, while–log(*P*-value) for association statistics is presented on the *y*-axis. The genome-wide significance threshold (*P* = 5×10^−8^) is depicted as a dotted horizontal line in **a.** The regional plots contain all genotyped and imputed SNPs in the region meta-analyzed between the discovery and replication cohorts.

In the combined analysis, two distinct genomic loci, on chromosomes 7p21.3 and Xq24, reached genome-wide significance (**Fig 2A**). The strongest association was located within a 200-kb interval on chromosome 7p21.3 (**Fig 2B**), explaining 4% of trait variance in Europeans and ~1% in East Asians (**S4 Table**). The only gene within this locus is *C1GALT1*, encoding core 1 synthase, glycoprotein-*N*-acetylgalactosamine 3-beta-galactosyltransferase 1. The top signal was represented by rs13226913 (*P* = 3.2x10^-11^), an intronic SNP within *C1GALT1*. This locus is further supported by rs1008897 (*P* = 9.1x10^-10^) in partial LD with rs13226913 (r^2^ = 0.33, D’ = 0.91 in Europeans and r^2^ = 0.52, D’ = 0.73 in East Asians). After mutual conditioning, both SNPs continue to be associated with the phenotype, suggesting a complex pattern of association at this locus (**S5 Table**).

The protein encoded by *C1GALT1* generates the common core 1 *O*-glycan structure by transferring galactose (Gal) from UDP-Gal to GalNAc-alpha-1-Ser/Thr. Core 1 *O*-glycans are the main glycans in the hinge region of circulating IgA1, as well as precursors of many extended mucin-type *O*-glycans on cell-surface and secreted glycoproteins. In humans, *C1GALT1* is abundantly expressed in IgA1-secreting cells[19], as well as in EBV-transformed lymphocytes, gastrointestinal tract, lungs, and kidneys[20]. The top SNP, rs13226913, is not in LD with any coding variant, but it perfectly tags several SNPs intersecting the ENCODE and Roadmap enhancers and promoters in immune cells, including EBV-immortalized B cells and primary CD19+ cells (**S6 Table**). Interrogation of eQTL databases revealed that rs13226913 has a highly significant cis-eQTL effect on *C1GALT1* in peripheral blood cells (*P* = 3.9 x 10^−23^) with the T allele associated with lower mRNA levels (**S7 Table**). Consistent with this finding, rs13226913 imparts an additive effect with each T (derived) allele increasing Gd-IgA1 levels by 0.22 standard deviation units (95% CI: 0.10–0.30).

The second genome-wide significant locus comprises a 100-kb interval on chromosome Xq24 (**Fig 2C**) and explains an additional 2.7% of the overall trait variance in Europeans and 1.2% in East Asians (**S4 Table**). The top signal at this locus is represented by rs5910940 (*P* = 2.7x10^-8^), a SNP 3’ downstream from *C1GALT1C1*. The T (derived) allele increases serum Gd-IgA1 levels by 0.14 standard deviation units per allele (95%CI: 0.11–0.17). Our post-hoc examination of genotypic effects suggests a dominant effect of the rs5910940-T allele in females (dominant model *P* = 7.9x10^-9^, **S8 Table**), although skewed inactivation of chromosome X in IgA1-producing cells could also potentially explain this effect.

*C1GALT1C1* encodes a transmembrane protein that is similar to the core 1 beta1,3-galactosyltransferase 1 encoded by *C1GALT1*. However, its gene product (known as COSMC) lacks the galactosyltransferase activity, and instead acts as a molecular chaperone required for the folding, stability, and full activity of C1GALT1[21]. *C1GALT1C1* is also ubiquitously expressed in multiple tissues, including IgA1-secreting cells[19], other blood cells, gastrointestinal tract, kidneys, and lungs[20]. Because sex chromosomes are not included in most eQTL analyses, we were not able to confirm if rs5910940 has an effect on the expression of *C1GALT1C1* based on available datasets. However, rs5910940 tags a 2-bp insertion in the active promoter of *C1GALT1C1* in B-lymphocytes and leukemia cell lines (**S9 Table**). Considering the known functional dependency of *C1GALT1* and *C1GALT1C1*, we also tested for potential epistasis between these two loci, but did not detect any significant genetic interaction.

**Fig 3:**
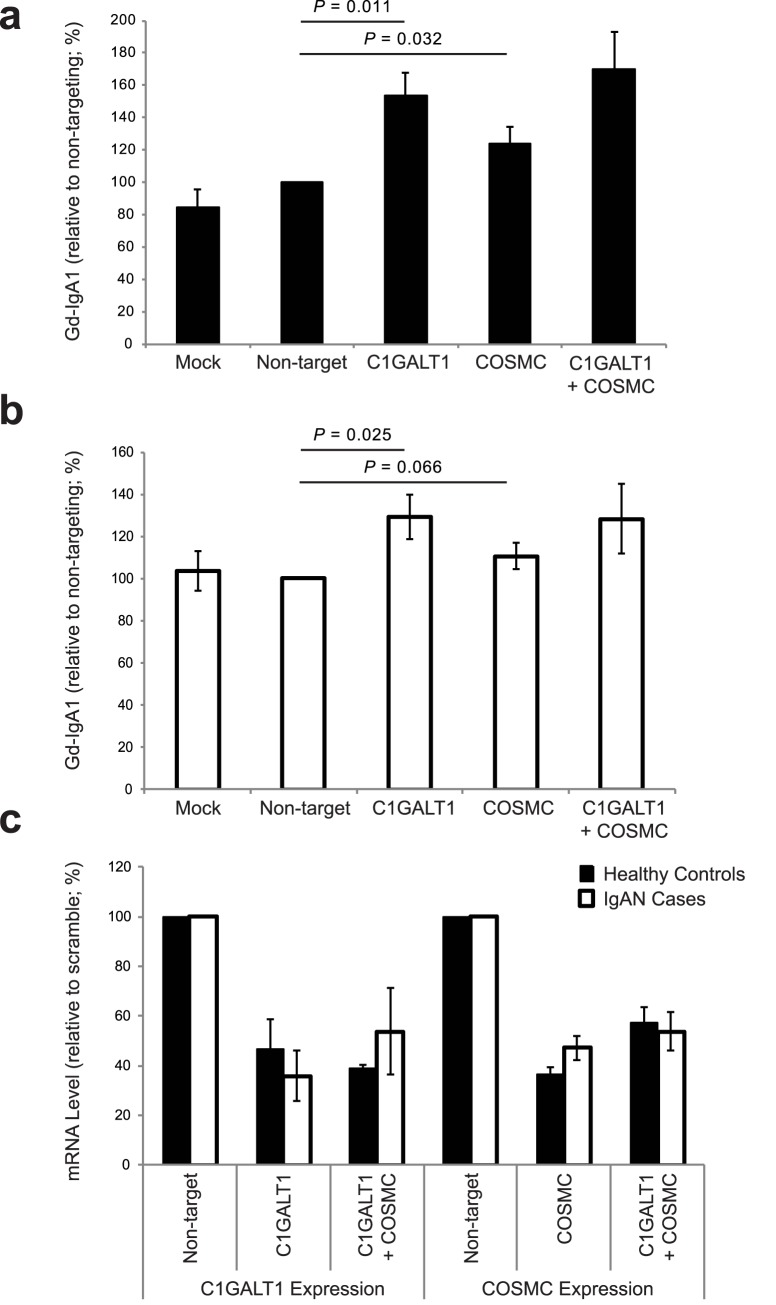
siRNA knock-down of C1GALT1, COSMC and COSMC+C1GALT1 in IgA1-secreting cell lines increases Gd-IgA1 production. **(a)** knock-down in IgA1-secreting cell lines from healthy controls; mock-control (n = 5), non-targeting siRNA (n = 7), C1GALT1 siRNA (n = 5), COSMC siRNA (n = 7), and COSMC+C1GALT1 siRNA (n = 2); **(b)** knock-down in IgA1-secreting cell lines from IgAN patients; mock-control (n = 5), non-targeting siRNA (n = 7), C1GALT1 (n = 5), COSMC siRNA (n = 7), and COSMC+C1GALT1 siRNA (n = 2); **(c)** relative change in mRNA in IgA1-secreting cell lines after siRNA knock-down of C1GALT1 (n = 5), COSMC (n = 7), and COSMC+C1GALT1 (n = 2) compared to non-targeting siRNA control.

Taken together, these data predict an additive regulatory effect of rs13226913 and rs5910940, resulting in lower *C1GALT1* and *C1GALT1C1* expression, and leading to increased production of Gd-IgA1. We next performed siRNA knock-down studies in human cultured IgA1-secreting cell lines to confirm the effect of lower *C1GALT1* and *C1GALT1C1* transcript abundance on the production of Gd-IgA1 (**Fig 3**). Consistent with the observed genetic effect, *in vitro* knock-down of *C1GALT1* resulted in 30–50% increased production of Gd-IgA1 by the cells derived from IgAN patients (*P* = 0.025) as well as from healthy controls (*P* = 0.011). Similar to *C1GALT1*, *in vitro* siRNA knock-down of *C1GALT1C1* in IgA1-producing cell lines significantly increased the production of Gd-IgA1 in healthy individuals (*P* = 0.032) and a similar trend was observed in IgAN patients (*P* = 0.066, **Fig 3**). Consistent with the genetic data, there was no multiplicative effect on Gd-IgA1 production with combined siRNA knock-down in IgA1-secreting lines.

**Fig 4:**
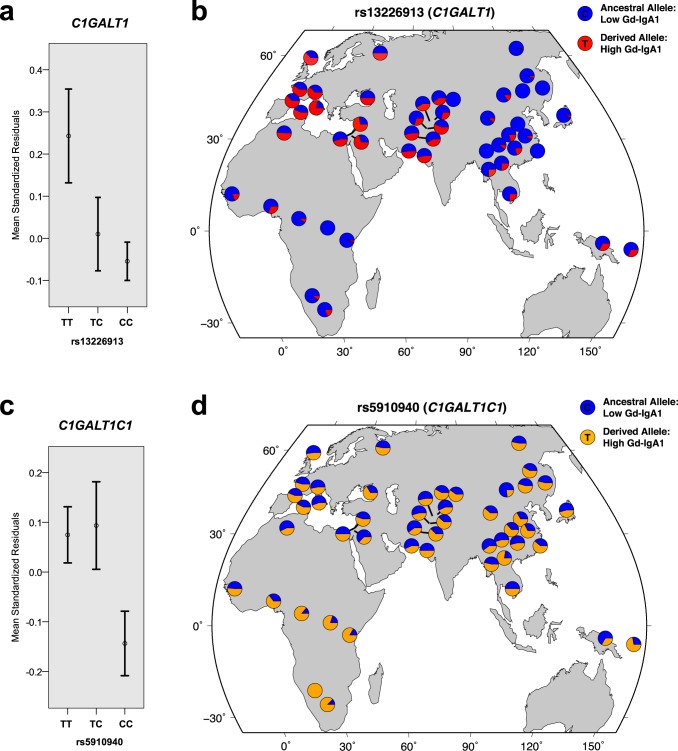
Genotypic effects and worldwide allelic frequency distribution for the two top genome-wide significant loci. **(a)** Mean trait values (+/- standard errors) by rs13226913 genotype at the *C1GALT1* locus. (**b**) The distribution of rs13226913 alleles across the Human Genome Diversity Panel (HGDP) populations. **(c)** Mean trait values (+/- standard errors) by rs5910940 genotype at the *C1GALT1C1* locus. **(d)** The distribution of rs5910940 alleles across HGDP populations. The allelic distribution plots were modified from the HGDP Selection Browser. The trait values were expressed as standard normal residuals of log-transformed serum Gd-IgA1 levels after adjustment for age, serum total IgA levels, case-control status and cohort membership.

Jointly, the newly discovered *C1GALT1* and *C1GALTC1* loci explain up to **7%** of variance in serum Gd-IgA1 levels in Europeans and **2%** in East Asians (**S4 Table**). Further examination of effect estimates by ethnicity confirms that the European cohorts predominantly drive these associations (**S10 Table**). Notably, the derived (T) allele of rs13226913 at *C1GALT1* locus is considerably more frequent in Europeans (freq. 47%) compared to East Asians (freq. 10%), additionally contributing to the difference in variance explained between ethnicities. Subsequent examination of allelic frequencies in the Human Genome Diversity Panel (**Fig 4**) confirms that the derived allele of rs13226913 is rare or absent in some Asian populations, while being the predominant (major) allele in Europeans (freq. ≥50%). In contrast, the T (derived) allele of rs5910940 at *C1GALT1C1* locus is equally frequent in Asian and European populations (freq. ~50%), but nearly fixed in selected African populations. These findings suggest potential involvement of geographically confined selective pressures acting on the loci controlling the *O*-glycosylation process.

Lastly, we detected additional suggestive signals, including a locus on chromosome 7p13 that warrants further follow-up in larger cohorts **(S2A and S2B Fig**). This locus is represented by rs978056 (*P* = 3.3x10^-5^), an intronic SNP in *HECW1* (encoding E3 ubiquitin ligase) previously studied in the context of colon and breast cancer **(S3 Fig**). Based on the analysis of known protein-protein interactions, HECW1 is a second-degree neighbor of C1GALT1 and COSMC, with ubiquitin C as a common interacting protein **(S2C Fig**).

## Discussion

Genetic studies of immune endophenotypes have provided novel insights into the genetic architecture of complex traits and enhanced sub-classification of several autoimmune and inflammatory disorders. The power of immune endophenotypes is best exemplified by recent genetic studies of ANCA titers in vasculitis[22], IgE levels in asthma[23, 24], and studies of IgG *N*-glycosylation and autoimmunity[18]. Taking a similar approach, we performed the first GWAS for aberrant *O*-glycosylation of IgA1.

Abnormalities in the *O*-glycan synthesis have been linked to several human diseases, including IgAN, inflammatory bowel disease (IBD), hematologic diseases, and cancer. Dense *O*-glycosylation of various mucins produced by epithelial cells is critical for the formation of a protective viscous barrier with anti-microbial properties at the mucosal surfaces of the gastrointestinal, urogenital and respiratory systems. Recent studies indicate that proper *O*-glycosylation of mucins is required for intestinal integrity in mice[25, 26] and may play a role in human susceptibility to IBD[27, 28]. In addition, *O*-glycosylation can affect the structure and immunogenicity of the modified proteins. For example, defective *O*-glycosylation represents the key pathogenic feature of Tn syndrome[29], where acquired enzymatic defect in the addition of galactose to *O*-glycans leads to exposed terminal GalNAc residue (Tn antigen) on the surface of red blood cells, triggering polyagglutination by naturally occurring anti-Tn antibodies[29]. Moreover, Tn and sialyl-Tn represent oncofetal antigens that are over-expressed in human cancers and may directly influence cancer growth, metastasis and survival, but the exact molecular perturbations that lead to *O*-glycosylation defects in tumor cells are presently not known[30].

Similar to Tn syndrome, the pathogenesis of IgAN involves autoimmune response to Tn antigens. In this case, the Tn antigen is exposed at the hinge region of IgA1 molecules as a result of aberrant *O*-glycosylation of IgA1 in the Golgi apparatus of IgA1-producing cells[9]. In patients with IgAN, the galactose-deficient IgA1 (Gd-IgA1) is recognized by circulating anti-Tn autoantibodies [5], leading to the formation of nephritogenic immune complexes[6–9]. Several independent studies, including in healthy twins and in families with IgAN, have demonstrated that serum levels of Gd-IgA1 have high heritability, providing high level of support for a genetic determination of this trait and a strong rationale for this study[12, 13, 31].

In this study, we quantified the levels of Gd-IgA1 in sera of 2,633 subjects of European and East-Asian ancestry using a simple lectin-based ELISA assay. Using GWAS approach, we discovered two genome-wide significant loci, on chromosomes 7p21.3 and Xq24, both with large effects on circulating levels of Gd-IgA1. The 7p21.3 locus contains *C1GALT1* gene, that encodes human core 1 β1–3-galactosyltransferase (C1GALT1), the key enzyme responsible for the addition of galactose to the Tn antigen. Mice deficient in C1GALT1 protein develop thrombocytopenia and kidney disease attributed to defective *O*-glycosylation of cell-surface proteins[32]. Moreover, C1GALT1 deficiency in mice results in a defective mucus layer, leading to spontaneous colonic inflammation that is dependent on the exposure to intestinal microbiota[25, 26]. C1GALT1 requires a molecular chaperone, COSMC, that ensures the enzyme is properly folded within the endoplasmic reticulum; loss of COSMC activity results in C1GALT1 being degraded in the proteosome[29]. Interestingly, COSMC is encoded by *C1GALT1C1* residing within our second genome-wide significant locus on chromosome Xq24. We also localized a suggestive locus on chromosome 7p13 that encodes an E3 ubiquitin ligase, but it is presently not known if this protein participates in the proteosomal degradation of C1GALT1. This signal will require further follow up. Importantly, our study demonstrates that there are several common genetic variants with relatively large effects on IgA1 *O*-glycosylation. These effects are conveyed by different genes, but converge on a single enzymatic step in the *O*-glycosylation pathway.

While we successfully identified two novel loci for serum Gd-IgA1 levels, several important limitations of our study design need to be acknowledged. First, our GWAS has a two-stage design and involves bi-ethnic cohorts. Although to date this is the largest study of individuals worldwide with measured serum Gd-IgA1 levels, this sample size is still not adequate to detect ethnicity-specific loci. Thus, our design is presently limited to the discovery of alleles that have similar effects in both Europeans and East Asians. At the same time, the bi-ethnic composition of our cohorts clearly enabled identification of the *C1GALT1* locus. The lead allele at this locus has a direction-consistent effect in both ethnicities, but because of the Gd-IgA1-increasing allele is relatively rare or even fixed in some Asian populations, this signal would have been missed if the discovery were performed entirely in East Asians. The second limitation relates to genomic resolution of our discovery study. Although genome-wide imputation was not performed at the time the study was conducted, post-hoc imputation using the latest 1000 Genomes reference revealed no additional loci outside of the regions that were originally selected for follow-up. Moreover, our conditional analyses revealed no additional signals among the imputed SNPs after controlling for the lead alleles at each locus, suggesting that our top SNPs captured most of the signal at the newly reported loci (**S4 Fig**).

Given the observed distributional differences in serum Gd-IgA1 levels between cases and controls (**S5 Fig**), we estimate that we would require a sample size of at least 24,000 cases and 24,000 controls to detect the effect of *C1GALT1* and *C1GALT1C1* loci in a bi-ethnic GWAS for IgAN (**S11 Table)**[33]. This sample size requirement is more than 3-fold greater than the largest bi-ethnic GWAS for IgAN published to date[34]. Moreover, considering weaker effect of these loci in East Asians, an even larger sample size (over 40,000 cases and 40,000 controls) would be required for a GWAS involving only East-Asian participants (5-fold greater than the largest published study for Chinese[35]). Yet, our endophenotype-based approach uncovered these loci in a minute fraction of the sample size required by a conventional case-control designs. Our power calculations also clearly indicate that much larger follow-up studies will be needed to conclusively demonstrate that Gd-IgA1-increasing alleles have a direct effect on the disease risk.

In summary, our results contribute new insights into the genetic regulation of *O*-glycan synthesis, and demonstrate that a simple lectin-based assay can be used effectively to map genetic regulators of *O*-glycosylation of serum proteins. Given the high heritability of this trait, it is likely that additional loci contribute to variation in Gd-IgA1 levels. In particular, the inheritance pattern in IgAN kindreds suggested segregation of a major dominant gene, indicating a potential role of additional rare alleles with large effects[12]. A search in larger population-based studies that includes both common and rare variants is likely to uncover additional genetic determinants of *O*-glycosylation defects and elucidate mechanisms leading to IgAN and related disorders.

## Materials and methods

### Study design

This study has a two-stage design (**S1 Fig**). In Stage 1 (the discovery phase) we performed a genome-wide meta-analysis of two discovery cohorts: the Chinese cohort of 950 individuals (483 cases and 467 controls, all Han Chinese ancestry, genotyped with Illumina 660-quad chip), and the US cohort of 245 individuals (141 cases and 104 controls, all European ancestry, genotyped with the Illumina 550v3 chip). Genome-wide scan was performed in both cohorts and fixed-effects meta-analysis was applied to prioritize signals for follow-up studies. In Stage 2 we performed targeted genotyping of the top signals from Stage 1 in five cohorts of European and East-Asian ancestry (1,438 individuals in total, **Table 1**). We estimated the power of our study design for a range of effect sizes under the following assumptions: standard normal trait distribution, additive risk model, no heterogeneity in association, marker allelic frequency of 0.25 (average MAF for the microarrays used), a follow-up significance threshold of P<5×10^−4^, and a combined significance level of P<5×10^−8^. These calculations demonstrate that we have adequate power to detect variants explaining ≥1.5% of overall trait variance (**S3 Table**). Our study was conducted according to the principles expressed in the Declaration of Helsinki; all subjects provided informed consent to participate in genetic studies, and the Institutional Review Board of Columbia University as well as local ethics review committees for each of the individual cohorts approved our study protocol.

### Phenotype measurements and quality control

The serum level of total IgA was determined using standard ELISA[36]. The serum level of Gd-IgA1 was determined using a custom HAA-based ELISA assay[12, 13, 36]. This method relies on the detection of HAA binding to desialylated galactose-deficient glycans (Tn antigens) of serum IgA1 immunocaptured on ELISA plates. Because in humans, IgA1, but not IgA2, has *O*-glycans, this assay effectively quantifies the serum level of Gd-IgA1 in units/ml. We have optimized this assay for high-throughput. Briefly, 96-well plates were coated with F(ab’)_2_ fragment of goat IgG anti-human IgA at 3 μg/ml. Plates were blocked with 1% BSA in PBS containing 0.05% Tween 20, and serial two-fold dilutions of samples and standards in blocking solution were incubated overnight at room temperature. To remove terminal sialic acid, the samples were treated with 100 μL (1 mU) per well of neuraminidase (Roche) in 10 mM sodium acetate buffer (pH = 5) for 3 h at 37°C. Next, the samples were incubated with GalNAc-specific biotinylated HAA lectin (Sigma-Aldrich) for 3 h at 37°C. The bound lectin was detected with avidin-horseradish peroxidase conjugate, followed by the peroxidase substrate, o-phenylenediamine-H_2_O_2_ (Sigma); the reaction was stopped with 1 M sulfuric acid. The concentration of Gd-IgA1 was calculated by interpolating the optical densities at 490 nm on calibration curves constructed using a myeloma Gd-IgA1 standard. The intra-assay coefficients of variation (CVs) for calibration curves, plotted by a 4-parameter model, ranged from 2–10% for the extremes of the curves and 1–5% in the middle region. If higher values were noted, the samples were re-analyzed. The inter-assay CV was also consistently under 5% and our prior studies demonstrated excellent reproducibility of this assay[36]. In the final analysis, we applied a correction for potential plate effects using the same replicate samples across all plates. After corrections, serum Gd-IgA1 levels for each cohort were tested for normality by the Shapiro-Wilk test, assisted by visual inspection of histograms and QQ-plots. Non-normal trait distributions were transformed using logarithmic transformation. The log-transformed traits were regressed against age and case-control status to derive standardized residuals. Summary statistics (mean, SD, skewness, and kurtosis) were derived for the distribution of standardized residuals, that were then used as a quantitative trait in GWAS analysis. Summary statistics, normality testing, transformations, plots, and regression analyses were performed with R 3.0 software package (CRAN).

### Stage 1: GWAS discovery

The discovery cohorts have been published, including details of the genotyping, genotype quality control, and ancestry analyses[34, 37]. Briefly, we implemented strict quality control analyses for each of the discovery cohorts, removing individual samples with low call rates, duplicates and samples with cryptic relatedness (pi-hat > 0.10), ancestry outliers, and samples with a detected sex mismatch. After all quality-control steps, the Chinese Discovery Cohort was composed of 950 individuals typed with 508,112 SNPs, while the US Discovery Cohort was composed of 245 individuals typed with 531,778 SNPs. In total, 468,781 SNPs overlapped between the cohorts, and this set of common markers was used for the discovery meta-analysis. To reduce any potential bias from population structure, we used modified PCA-based ancestry matching algorithms (Spectral-GEM software)[38, 39], as described in our prior studies of these cohorts[34, 37]. Primary association testing for the Gd-IgA1 phenotype (expressed as standardized residuals) was performed for each individual cohort under an additive linear model in PLINK[40]. We included significant principal components of ancestry as covariates in linear models used for association testing. Additionally, we performed regression analyses with and without adjustment for serum total IgA levels. We derived adjusted effect estimates with standard errors for each SNP, and we combined these results using an inverse variance-weighted method (METAL software)[41]. We visually examined genome-wide distributions of *P*-values using QQ-plots for each individual cohort, as well as for the joint analysis of both cohorts. We estimated the genomic inflation factors[42], that were negligible for each individual discovery cohort (lambda = 1.011 and 1.013 for the Chinese and US cohorts, respectively). The overall genomic inflation factor was estimated at 1.010 and the final meta-analysis QQ-plots showed no global deviation from the expected distribution of *P*-values (**S1 Fig**).

### Stage 2: Follow-up of suggestive signals

We next prioritized the top 50 SNPs for replication among the top suggestive SNPs with P<5x10^-4^ from the GWAS analyses. First, we clustered the top signals into distinct loci based on their genomic coordinates and metrics of LD. Conditional regression analysis was carried out to detect independent association between signals within the same genomic regions. For genotyping in replication cohorts, we prioritized the independent SNPs that had the lowest P-value at each genomic locus. In addition, we required that each SNP be successfully genotyped in both discovery cohorts. We excluded 'singleton signals' defined as loci supported by only a single SNP in the absence of supporting signals with P<0.01 within the same LD block. If the genotyping assay failed for the top SNP, a back-up SNP was selected on the basis of its strength of phenotypic association, LD with the top SNP, genotyping quality, and ability to successfully design working primers. Moreover, we added SNPs for which the signals became more significant (P<5x10^-4^) after adjustment for serum total IgA levels. In all, we successfully genotyped 50 carefully selected SNPs in 1,438 independent replication samples across five cohorts. Similar to our prior studies, the genotyping of replication cohorts was performed using KASP (Kompetitive Alelle Specific PCR, LGC Genomics). In our prior studies, this technology had >99.8% accuracy rates[43]. **Table 1** summarizes the ethnic composition of our replication cohorts along with the genotyping method and average genotype call rates. We first carried out association analyses individually within each of the cohorts using the same methods as in the discovery study. Next, we combined the results using a fixed-effects model (**S2 Table**). For each of the genotyped SNPs, we derived pooled effect estimates and their 95% confidence intervals. To declare genome-wide significance, we used the generally accepted threshold of P<5x10^-8^, initially proposed for Europeans genotyped with high-density platforms based on extrapolation to infinite marker density[44].

### Chromosome X analysis

We performed two types of association tests for X-linked markers. Our primary association test involved sex-stratified meta-analysis of chromosome X markers: each male and female sub-cohort was analyzed separately and the association statistics were combined across all sub-cohorts using fixed effects meta-analysis. This approach is not affected by the type of allele coding in males and allows for different effect size estimates between males and females**[27]**. In secondary analyses, we assumed complete X-inactivation in females and a similar effect size between males and females. In this test, females are considered to have 0, 1, or 2 copies of an allele as in an autosomal analysis while males are considered to have 0 or 2 copies of the same allele (*i*.*e*., male hemizygotes are equivalent to female homozygotes). The main limitation of this approach relates the assumption of complete X inactivation. Because approximately 15–25% of X-linked genes escape inactivation in female-derived fibroblasts[45] and chromosome X inactivation has not been studied in IgA1-secreting cells, this analysis was performed only on an exploratory basis, but the results were consistent with sex-stratified analyses.

### Tests of alternative inheritance models and epistasis

For the genome-wide significant loci, we explored two alternative genetic models (dominant and recessive) and compared these models using Bayesian Information Criterion (**S8 Table**). We also tested for all pairwise genetic interactions between the suggestive and significant loci using two different tests. First, we used a 1-degree of freedom likelihood ratio test to compare two nested linear regression models: the model with main effects only versus the model with main effects plus additive interaction terms. Second, a more general 4-degree of freedom genotypic interaction test was performed. In this test, we compared a model with allelic effects, dominant effects, and their interaction terms with a reduced model without any of the interaction terms. All models were stratified by sex and cohort. The analyses were performed in R 3.0 software package (CRAN).

### Imputation and conditional analyses of significant and suggestive loci

To interrogate any potential SNPs that were not directly typed in our dataset, we downloaded the latest release of the 1000 Genomes (Phase 3) and imputed our discovery cohorts using ethnicity-specific reference panels. The haplotypes were phased using Markov Chain Haplotyping software (MACH) and the imputations were carried out with Minimac3. For downstream analyses, we applied strict quality control filters post-imputation, including only markers that were either genotyped or imputed with high confidence (R2 ≥ 0.8). Association testing of imputed SNPs was performed assuming an underlying additive linear model and including cohort-specific significant principal components as covariates. Primary analysis was performed using a dosage association method in PLINK, that accounts for uncertainty in prediction of the imputed data by weighting genotypes by their posterior probabilities. We used a similar approach to perform conditional analyses across the top loci, with conditioning SNPs added as additional covariates in linear models.

### Functional annotation of significant and suggestive loci

Using the imputed results for the *C1GALT1*, *C1GALT1C1*, and *HECW1* regions, we examined all of the top most associated variants as well as all SNPs in LD with the lead SNP (r2>0.5) at each locus. We annotated these variants using ANNOVAR[46], SeattleSeq[47], SNPNexus[48], FunciSNP[49], HaploReg4[50], and ChroMos[51]. The transcripts whose expressions were correlated with the lead SNPs in cis- or trans- were also identified using available eQTL datasets, including: (1) peripheral blood eQTLs based on meta-analysis of 5,311 Europeans[52], (2) primary B-cell and monocyte eQTLs from 288 Europeans[53], and (3) the latest release of GTEx data across multiple tissue types[20, 54]. We utilized, automated MEDLINE text mining tools to assess network connectivity between genes residing in implicated GWAS loci, including GRAIL[55], e-LiSe[56], and FACTA+[57]. We also interrogated all known protein-protein interaction networks for connectivity between candidate genes using the Disease Association Protein-Protein Link Evaluator (DAPPLE)[58] and Protein Interaction Network Analysis platform (PINA2)[59]. We used Cytoscape v.2.8 to visualize network graphs.

### siRNA knock-down studies in IgA1 secreting cell lines

IgA1-secreting cell lines from five patients with IgAN and five healthy controls were transfected using ON-TARGETplus SMARTpool siRNAs (Thermo Fisher Scientific) specific for human *C1GALT1*, *COSMC*, or both. The ON-TARGETplus Non-targeting Pool siRNAs was used as a control. We followed our previously published protocol for Amaxa nucleofector II (Lonza)[60]. Twenty-four hours after transfection, the knock-down efficiency was determined by qRT-PCR with previously described primers[9, 60]. The knockdown was expressed as cDNA level of the individual gene normalized to GAPDH after respective siRNA treatment, divided by the respective value obtained after treatment by non-targeting siRNA. The effect of siRNA knock-down on the phenotype (the degree of galactose-deficiency of IgA1) was based on the reactivity of secreted IgA1 with a lectin from *Helix aspersa* specific for terminal GalNAc, as described[9, 60].

## Supporting information

S1 Fig Study design and quantile-quantile plots for the discovery meta-analysis. **(a)** Study flowchart summarizing the discovery cohorts (stage 1) and the replication cohorts (stage 2) with final numbers of individuals after phenotype and genotype quality control analyses; **(b)** QQ-plot for the genome-wide discovery meta-analysis (N = 1,195) of serum Gd-IgA1 levels without adjustment for serum total IgA levels and **(c)** after adjustment for serum total IgA levels. All signals with P<5x10^-4^ (horizontal line) from both analyses were prioritized for follow-up in replication cohorts (stage 2). Lambda: genomic inflation factor.
(PDF)

S2 Fig The suggestive locus on chromosome 7p13 encoding *HECW1*. (**a**) Mean trait values (+/- standard errors) by rs978056 genotype. **(b)** Regional plot of the *HECW1* locus and the top signal represented by rs978056 (P = 3.3x10^-5^); the *x*-axis presents physical distance in kilobases (hg18 coordinates), and the *y*-axis presents −log *P* values for association statistics. **(c)** The network of known protein-protein interactions between *HECW1*, *C1GALT1*, and *C1GALT1C1*-encoded proteins. Each node represents a protein and each edge represents a high confidence physical interaction. The seed terms are highlighted in green and their common interactors in yellow. The protein interactions were analyzed and visualized using the Protein Interaction Network Analysis (PINA2) platform.
(PDF)

S3 Fig The gene-phenotype co-citation network. The co-citation network was constructed based on all PubMed abstracts for the query terms C1GALT1 (61 abstracts), C1GALT1C1 (39 abstracts), and HECW1 (5 abstracts). Both human and mouse disease phenotypes (circles) were analyzed for co-citation (edges) with the three query terms (green diamonds). Common interactors are highlighted in yellow. The PubMed query was performed on December 15^th^, 2015 and the gene-phenotype network was visualized in Cytoscape v.2.8. IgAN: IgA nephropathy; HSPN: Henoch-Schoenlein purpura nephritis; ALS: amyotrophic lateral sclerosis.
(PDF)

S4 Fig Conditional analysis of the top three loci using all imputed markers (1000 Genomes reference, version 3). The top row depicts unconditioned discovery meta-analysis results for all the imputed markers at the **(a)** *C1GALT1*, **(b)** *C1GALT1C1*, and **(c)** *HECW1* loci. The bottom row depicts the discovery meta-analysis results after conditioning individual cohort results for the lead SNP(s) at each locus: **(d)** rs13226913 and rs1008897 at the *C1GALT1* locus, **(e)** rs5910940 and rs2196262 at the *C1GALT1C1* locus, and **(f)** rs978056 at the *HECW1* locus. The red dotted line corresponds to *P* = 1 x 10^−3^ and is provided for reference.
(PDF)

S5 Fig Density plots for the distribution of adjusted and standardized Gd-IgA1 levels by case/control status. The distributional differences in Gd-IgA1 levels between cases and controls for **(a)** all study cohorts, **(b)** European cohorts, and **(c)** East Asian cohorts. The Gd-IgA1 trait is expressed as standardized residuals of natural log-transformed serum Gd-IgA1 levels after adjustment for age, sex, total IgA levels, and cohort membership; each standard deviation increase in the Gd-IgA1 endophenotype is associated with disease OR (95% CI) of 1.53 (1.40–1.68), 1.49 (1.31–1.72), and 1.56 (1.37–1.78) for All, European, and East Asian cohorts, respectively.
(PDF)

S1 Table Association of known IgAN susceptibility loci with serum Gd-IgA1 levels in the joint analysis of the discovery cohorts (total N = 1,195). The association results were adjusted for age, total IgA, case-control status, ancestry, and cohort membership.
(PDF)

S2 Table Combined association results for the 50 loci selected for replication. Serum Gd-IgA1 levels before and after adjustment for serum total IgA levels.
(PDF)

S3 Table Study power. The power was estimated for a range of effect sizes expressed as fraction of total variance of the quantitative trait explained by a genetic variant (columns). The assumptions include: standard normal trait distribution, additive risk model, no heterogeneity, marker allelic frequency of 0.25, perfect LD between a marker and a causal allele, a follow-up significance threshold of P<5×10^−4^ (top row) and a joint significance level of P<5×10^−8^ (bottom row). Shaded in red is the study detection limit corresponding to alleles explaining 1.5% of total variance.
(PDF)

S4 Table Total variance explained by genome-wide significant loci. The fraction of total variance explained was estimated by regressing individual genetic predictors (additive coding) against the outcome of standardized residuals for the trait (Gd-IgA1 levels adjusted for age, case-control status, and serum total IgA levels) and deriving R^2^ for the regression model. The total variance explained across multiple cohorts was calculated as an average fraction of explained variance for individual cohorts weighted by cohort size. The variance explained by the *C1GALT1* locus was calculated by including both rs13226913 and rs1008897 in the regression model. For *C1GALT1C1* locus, both rs5910940 and rs2196262 were included under additive coding. The total variance explained jointly by *C1GALT1* and *C1GALT1C1* loci was calculated by including all four SNP predictors from these loci in a single regression model.
(PDF)

S5 Table Mutual conditioning across the genome-wide significant loci. Each SNP that reached genome-wide significance in our study was conditioned on all other SNPs that reached genome-wide significance, one at a time. Highlighted in red are independent effects for markers located within the same locus after conditioning on the other significant marker within the same locus. Notably, conditioning within each locus demonstrates residual effects, while mutual conditioning across loci strengthens the association signal at each locus. Because chromosome X markers are included in these analyses, all models were sub-stratified based on sex; the conditioning was first performed within each sub-cohort, then the results were combined using fixed effects meta-analysis. In all analyses, markers were coded under an additive model and the Gd-IgA1-increasing allele was used as a test allele. StdErr. Standard error.
(PDF)

S6 Table HaploReg regulatory annotations for variants in linkage disequilibrium (r2<0.85) with rs13226913 based on Roadmap Epigenomes and ENCODE data: sorted by r^2^ with rs13226913; most promising candidates highlighted in red. (XLSX)

S7 Table Expression QTL effects of rs13226913 across multiple tissue types. (PDF)

S8 Table Exploration of alternative genetic models. We explored two alternative genetic models (dominant and recessive) and compared these models using Bayesian Information Criterion (BIC). The best model is highlighted in red. While this analysis suggests an additive model for 4 out of 5 top markers, the effect of rs5910940 (*C1GALT1C1* locus) is best explained by a T-allele dominant model. All analyses were stratified based on sex, explaining slight differences in effect estimates and p-values compared to Table 2. StdErr: standard error.
(PDF)

S9 Table HaploReg regulatory annotations for variants in linkage disequilibrium (r2<0.85) with rs5910940 based on Roadmap Epigenomes and ENCODE data: sorted by r^2^ with rs5910940; most promising candidates highlighted in red. (XLSX)

S10 Table Ethnicity-specific association results for the significant and suggestive loci. The East Asians include the Chinese Discovery, the Chinese Replication, and the Japanese Replication cohorts (N = 1,603). The Europeans include the US discovery cohort (100% self-identified Whites), German, French, and US Replication cohorts (N = 1,030). The results for all ethnicity-defined cohorts were combined using fixed effects meta-analysis. Allelic frequencies were averaged within the ethnicity-defined cohorts.
(PDF)

S11 Table Sample sizes required for testing new Gd-IgA1 loci for association with IgA nephropathy. Minimum sample sizes (cases + controls) required to detect associations of the newly detected Gd-IgA1 loci with the risk of IgAN in East Asian, European, and bi-ethnic GWAS assuming observed effect sizes, 50% case proportion, α = 5 x 10^−8^, and power (1-β) of 80%, 90% and 99.9%. The variance explained by each locus was derived as in S4 Table. The observed ORs of disease per standard deviation of endophenotype were calculated based on logistic regression with case/control status as an outcome and standardized residuals of Gd-IgA1 (after adjustment for age, sex, cohort, and total IgA levels) as a predictor. Separate estimates were obtained for our East Asian, European, and bi-ethnic cohorts. The calculations were performed within the framework of Mendelian Randomization, as previously proposed by Brion et al. *Int J Epidemiol* 42,1497–501 (2013) and implemented in the online calculator at https://cnsgenomics.shinyapps.io/mRnd/
(PDF)
