# Supplementary material for: GWAS for serum galactose-deficient IgA1 implicates critical genes of the O-glycosylation pathway
Source: PLoS Genet. 2017 Feb 10;13(2):e1006609. doi: 10.1371/journal.pgen.1006609 (PMC5328405; doi:10.1371/journal.pgen.1006609)
Supplement: S1 Fig — (a) Study flowchart summarizing the discovery cohorts (stage 1) and the replication cohorts (stage 2) with final numbers of individuals after phenotype and genotype quality control analyses; (b) QQ-plot for the genome-wide discovery meta-analysis (N = 1,195) of serum Gd-IgA1 levels without adjustment for serum total IgA levels and (c) after adjustment for serum total IgA levels. All signals with P<5x10^-4^ (horizontal line) from both analyses were prioritized for follow-up in replication cohorts (stage 2). Lambda: genomic inflation factor. (PDF) [file pgen.1006609.s001.pdf]

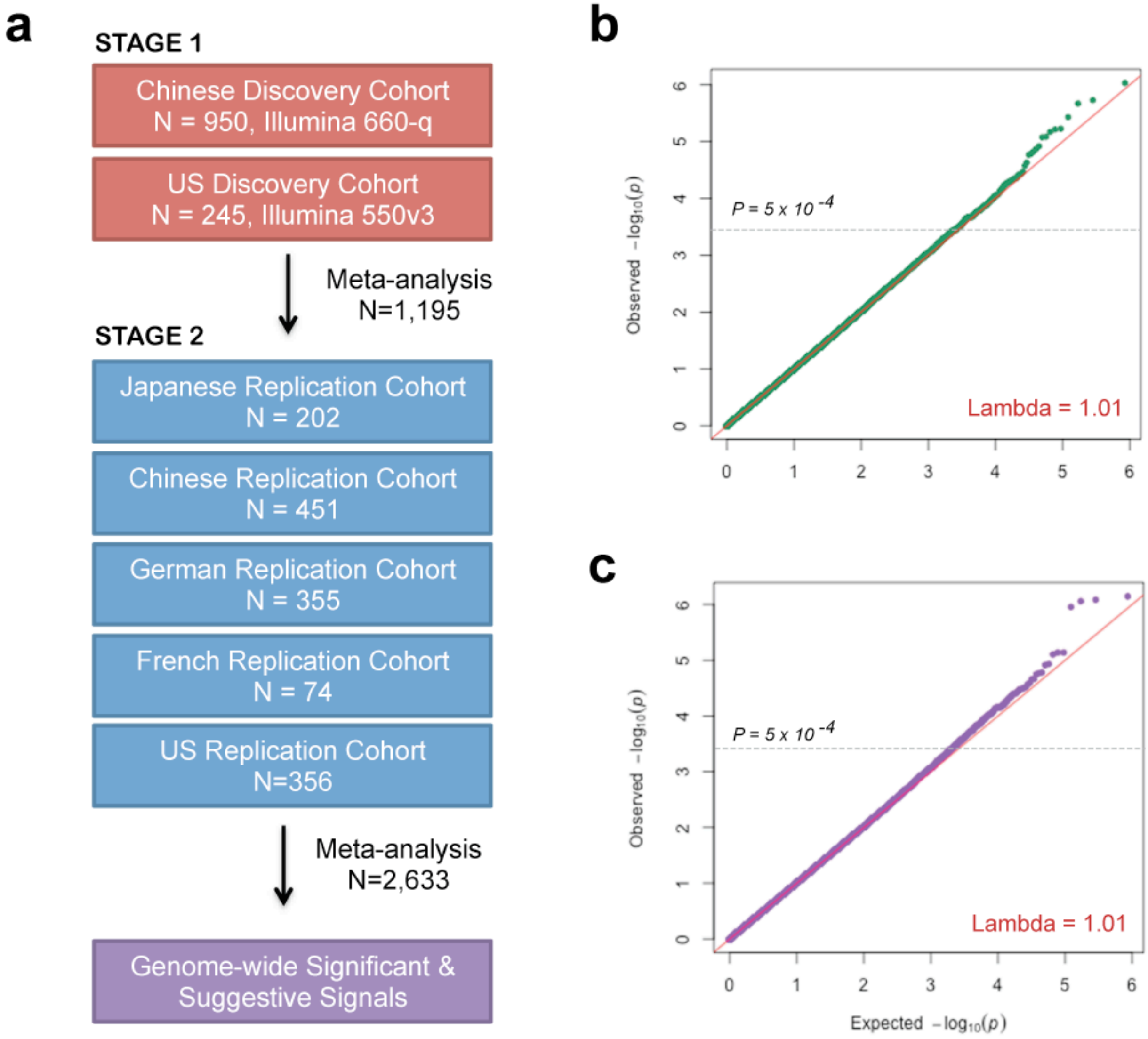


**Supplementary Figure 1**

**Study design and quantile-quantile plots for the discovery meta-analysis**

**(a)** Study flowchart summarizing the discovery cohorts (stage 1) and the replication cohorts (stage 2) with final numbers of individuals after phenotype and genotype quality control analyses; **(b)** QQ-plot for the genome-wide discovery meta-analysis (N=1,195) of serum Gd-IgA1 levels without adjustment for serum total IgA levels and **(c)** after adjustment for serum total IgA levels. All signals with $P<5\times10^{-4}$ (horizontal line) from both analyses were prioritized for follow-up in replication cohorts (stage 2). Lambda: genomic inflation factor.
