# Supplementary material for: GWAS for serum galactose-deficient IgA1 implicates critical genes of the O-glycosylation pathway
Source: PLoS Genet. 2017 Feb 10;13(2):e1006609. doi: 10.1371/journal.pgen.1006609 (PMC5328405; doi:10.1371/journal.pgen.1006609)
Supplement: S2 Fig — (a) Mean trait values (+/- standard errors) by rs978056 genotype. (b) Regional plot of the HECW1 locus and the top signal represented by rs978056 (P = 3.3x10^-5^); the x-axis presents physical distance in kilobases (hg18 coordinates), and the y-axis presents −log P values for association statistics. (c) The network of known protein-protein interactions between HECW1, C1GALT1, and C1GALT1C1-encoded proteins. Each node represents a protein and each edge represents a high confidence physical interaction. The seed terms are highlighted in green and their common interactors in yellow. The protein interactions were analyzed and visualized using the Protein Interaction Network Analysis (PINA2) platform. (PDF) [file pgen.1006609.s002.pdf]

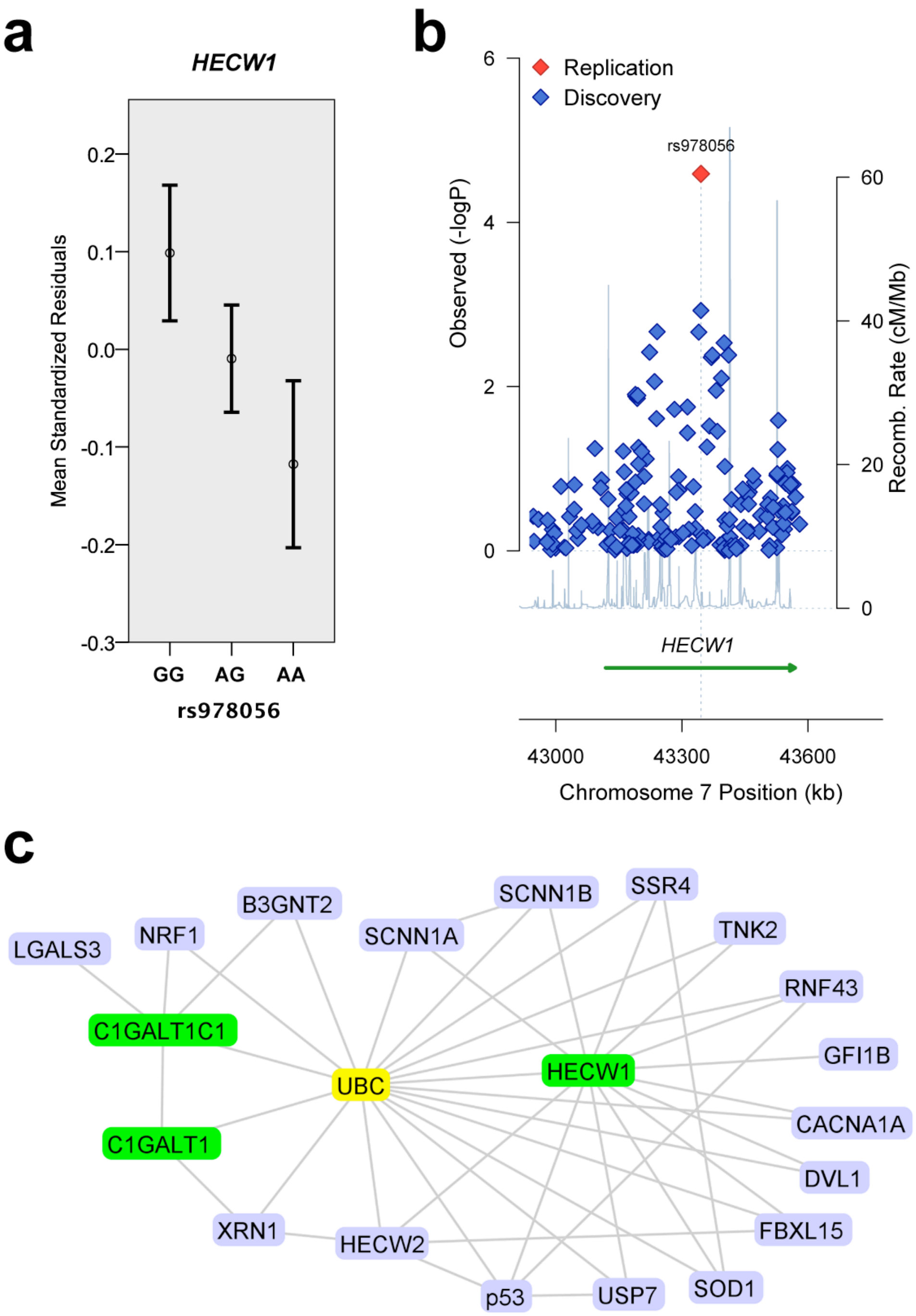


**Supplementary Figure 2**

**The suggestive locus on chromosome 7p13 encoding *HECW1*.**

(**a**) Mean trait values (+/- standard errors) by rs978056 genotype. **(b)** Regional plot of the *HECW1* locus and the top signal represented by rs978056 ($P=3.3x10^{-5}$); the *x*-axis presents physical distance in kilobases (hg18 coordinates), and the *y*-axis presents −log *P* values for association statistics. **(c)** The network of known protein-protein interactions between *HECW1*, *C1GALT1*, and *C1GALT1C1*-encoded proteins. Each node represents a protein and each edge represents a high confidence physical interaction. The seed terms are highlighted in green and their common interactors in yellow. The protein interactions were analyzed and visualized using the Protein Interaction Network Analysis (PINA2) platform.
