# Supplementary material for: GWAS for serum galactose-deficient IgA1 implicates critical genes of the O-glycosylation pathway
Source: PLoS Genet. 2017 Feb 10;13(2):e1006609. doi: 10.1371/journal.pgen.1006609 (PMC5328405; doi:10.1371/journal.pgen.1006609)
Supplement: S3 Fig — The co-citation network was constructed based on all PubMed abstracts for the query terms C1GALT1 (61 abstracts), C1GALT1C1 (39 abstracts), and HECW1 (5 abstracts). Both human and mouse disease phenotypes (circles) were analyzed for co-citation (edges) with the three query terms (green diamonds). Common interactors are highlighted in yellow. The PubMed query was performed on December 15th, 2015 and the gene-phenotype network was visualized in Cytoscape v.2.8. IgAN: IgA nephropathy; HSPN: Henoch-Schoenlein purpura nephritis; ALS: amyotrophic lateral sclerosis. (PDF) [file pgen.1006609.s003.pdf]

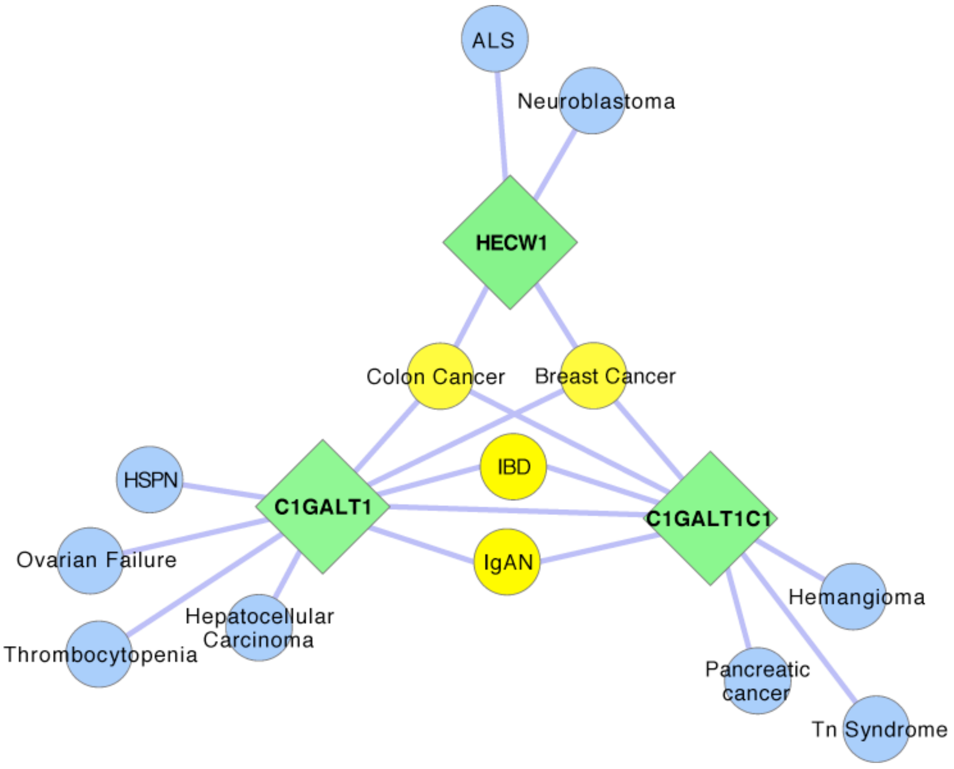

Supplementary Figure 3

The gene-phenotype co-citation network.

The co-citation network was constructed based on all PubMed abstracts for the query terms C1GALT1 (61 abstracts), C1GALT1C1 (39 abstracts), and HECW1 (5 abstracts). Both human and mouse disease phenotypes (circles) were analyzed for co-citation (edges) with the three query terms (green diamonds). Common interactors are highlighted in yellow. The PubMed query was performed on December 15th, 2015 and the gene-phenotype network was visualized in Cytoscape v.2.8. IgAN: IgA nephropathy; HSPN: Henoch-Schoenlein purpura nephritis; ALS: amyotrophic lateral sclerosis.
