# Supplementary material for: GWAS for serum galactose-deficient IgA1 implicates critical genes of the O-glycosylation pathway
Source: PLoS Genet. 2017 Feb 10;13(2):e1006609. doi: 10.1371/journal.pgen.1006609 (PMC5328405; doi:10.1371/journal.pgen.1006609)
Supplement: S4 Fig — The top row depicts unconditioned discovery meta-analysis results for all the imputed markers at the (a) C1GALT1, (b) C1GALT1C1, and (c) HECW1 loci. The bottom row depicts the discovery meta-analysis results after conditioning individual cohort results for the lead SNP(s) at each locus: (d) rs13226913 and rs1008897 at the C1GALT1 locus, (e) rs5910940 and rs2196262 at the C1GALT1C1 locus, and (f) rs978056 at the HECW1 locus. The red dotted line corresponds to P = 1 x 10^−3^ and is provided for reference. (PDF) [file pgen.1006609.s004.pdf]

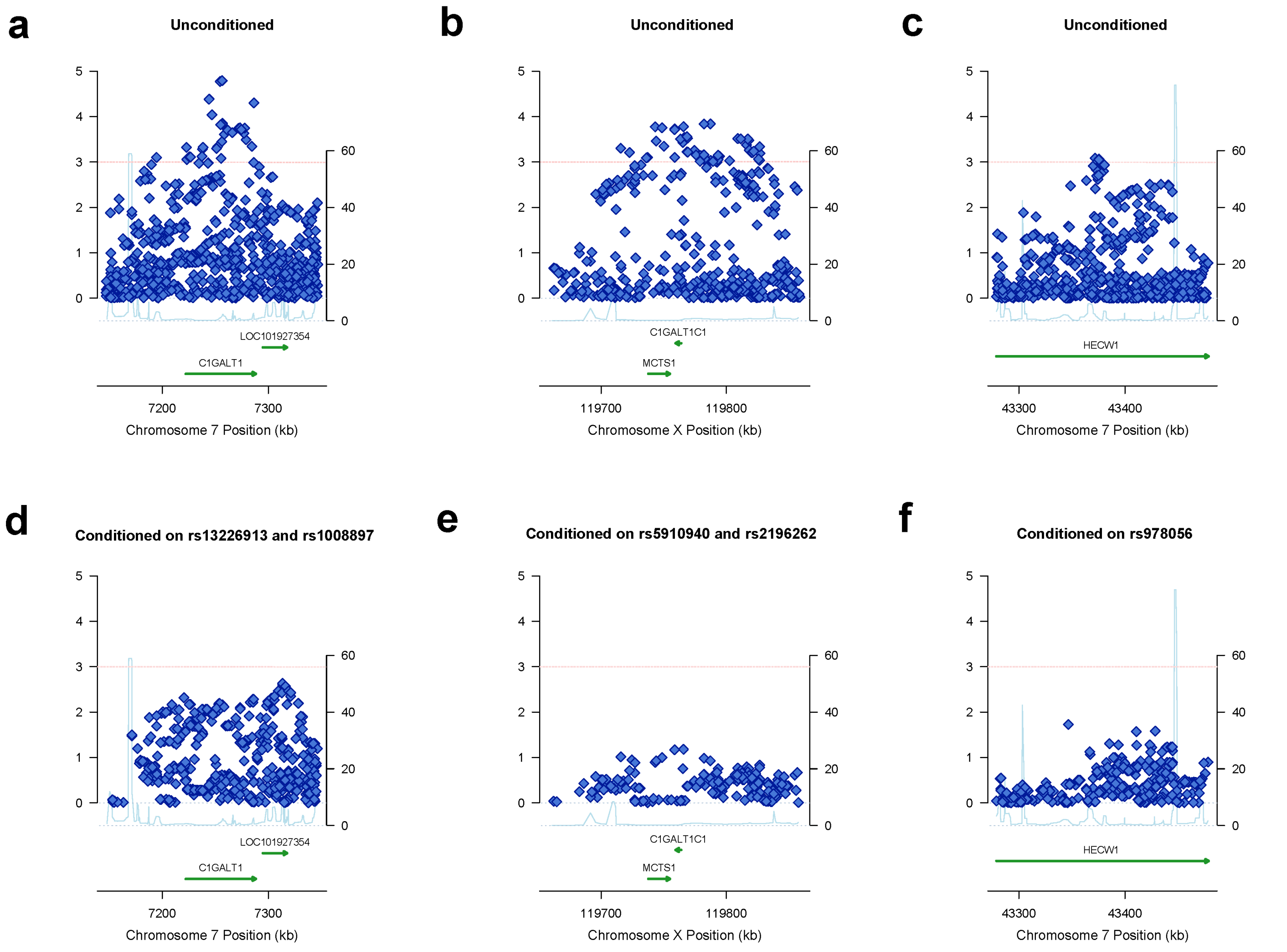

Supplementary Figure 4.

Conditional analysis of the top three loci using all imputed markers (1000 Genomes reference, version 3).

The top row depicts unconditioned discovery meta-analysis results for all the imputed markers at the **(a)** *C1GALT1*, **(b)** *C1GALT1C1*, and **(c)** *HECW1* loci. The bottom row depicts the discovery meta-analysis results after conditioning individual cohort results for the lead SNP(s) at each locus: **(d)** rs13226913 and rs1008897 at the *C1GALT1* locus, **(e)** rs5910940 and rs2196262 at the *C1GALT1C1* locus, and **(f)** rs978056 at the *HECW1* locus. The red dotted line corresponds to $P = 1 \times 10^{-3}$ and is provided for reference.
