# Supplementary material for: GWAS for serum galactose-deficient IgA1 implicates critical genes of the O-glycosylation pathway
Source: PLoS Genet. 2017 Feb 10;13(2):e1006609. doi: 10.1371/journal.pgen.1006609 (PMC5328405; doi:10.1371/journal.pgen.1006609)
Supplement: S5 Fig — The distributional differences in Gd-IgA1 levels between cases and controls for (a) all study cohorts, (b) European cohorts, and (c) East Asian cohorts. The Gd-IgA1 trait is expressed as standardized residuals of natural log-transformed serum Gd-IgA1 levels after adjustment for age, sex, total IgA levels, and cohort membership; each standard deviation increase in the Gd-IgA1 endophenotype is associated with disease OR (95% CI) of 1.53 (1.40–1.68), 1.49 (1.31–1.72), and 1.56 (1.37–1.78) for All, European, and East Asian cohorts, respectively. (PDF) [file pgen.1006609.s005.pdf]

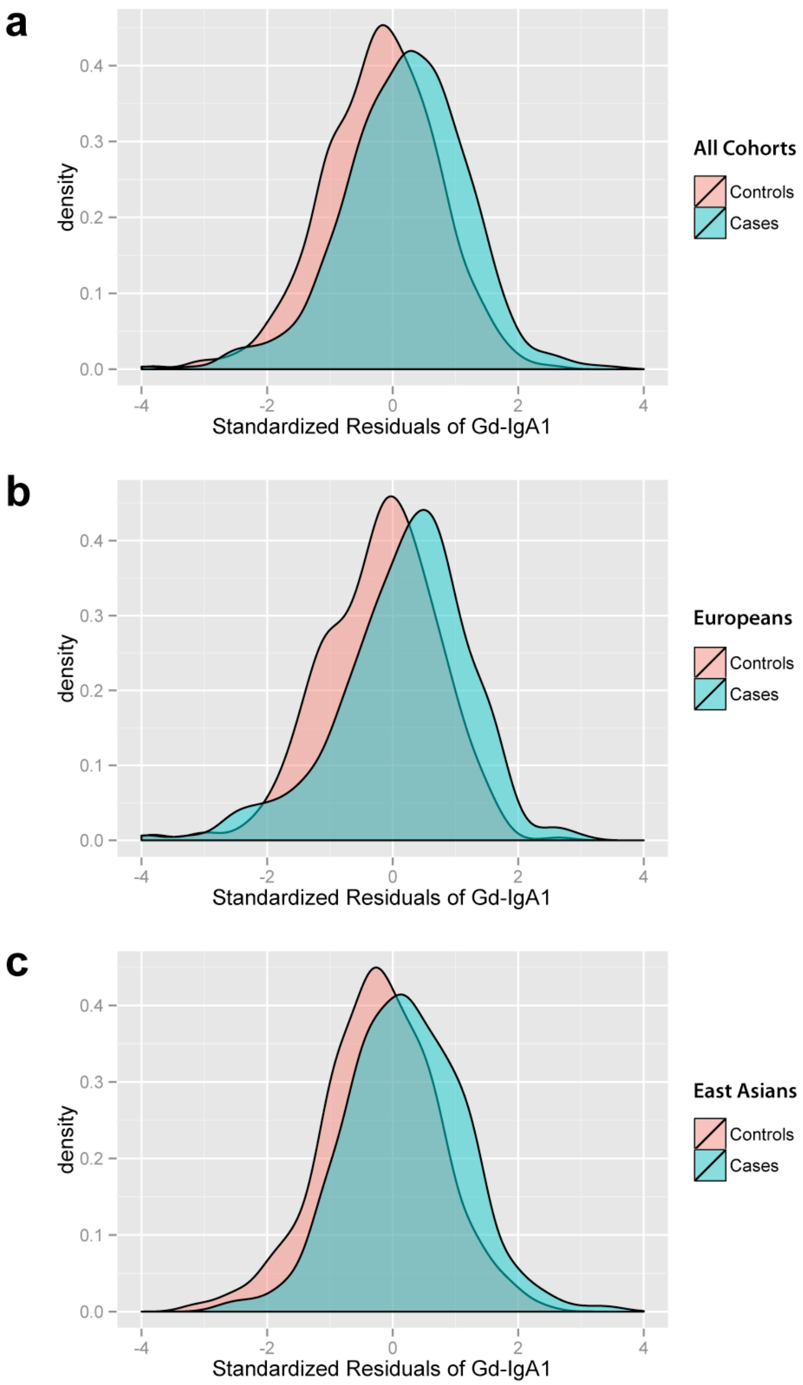

Supplementary Figure 5

Density plots for the distribution of adjusted and standardized Gd-IgA1 levels by case/control status

The distributional differences in Gd-IgA1 levels between cases and controls for **(a)** all study cohorts**, (b)** European cohorts, and **(c)** East Asian cohorts. The Gd-IgA1 trait is expressed as standardized residuals of natural log-transformed serum Gd-IgA1 levels after adjustment for age, sex, total IgA levels, and cohort membership; each standard deviation increase in the Gd-IgA1 endophenotype is associated with disease OR (95% CI) of 1.53 (1.40-1.68), 1.49 (1.31-1.72), and 1.56 (1.37-1.78) for All, European, and East Asian cohorts, respectively.
