# Supplementary material for: GWAS for serum galactose-deficient IgA1 implicates critical genes of the O-glycosylation pathway
Source: PLoS Genet. 2017 Feb 10;13(2):e1006609. doi: 10.1371/journal.pgen.1006609 (PMC5328405; doi:10.1371/journal.pgen.1006609)
Supplement: S1 Table — The association results were adjusted for age, total IgA, case-control status, ancestry, and cohort membership. (PDF) [file pgen.1006609.s006.pdf]

Supplementary Table 1. Association of known IgAN susceptibility loci with serum Gd-IgA1 levels in the joint analysis of the discovery cohorts (total N=1,195): The association results were adjusted for age, total IgA, case-control status, ancestry, and cohort membership.

Chr	SNP	IgAN risk allele	Frequency	Effect on Gd-IgA1	StdErr	P-value	Genes in Locus
1	rs17019602	G	0.80	0.02	0.04	0.60 (NS)	*VAV3*
1	rs6677604	G	0.09	0.06	0.06	0.28 (NS)	*CFHR3-CFHR1* deletion
6	rs7763262	C	0.26	0.03	0.04	0.40 (NS)	*HLA-DR–HLA-DQ*
6	rs9275224	G	0.40	0.04	0.03	0.14 (NS)	*HLA-DR–HLA-DQ*
6	rs2856717	G	0.25	0.05	0.03	0.17 (NS)	*HLA-DR–HLA-DQ*
6	rs9275596	T	0.21	0.04	0.04	0.21 (NS)	*HLA-DR–HLA-DQ*
6	rs2071543	G	0.18	-0.08	0.04	0.03 (NS)	*TAP2-PSMB9*
6	rs1883414	G	0.22	0.01	0.04	0.74 (NS)	*HLA-DP*
8	rs2738048	T	0.29	0.02	0.03	0.49 (NS)	*DEFA*
8	rs10086568	A	0.70	0.01	0.03	0.83 (NS)	*DEFA*
9	rs4077515	T	0.68	0.05	0.03	0.11 (NS)	*CARD9*
16	rs11150612	A	0.30	0.01	0.03	0.76 (NS)	*ITGAM-ITGAX*
16	rs11574637	T	0.17	0.02	0.12	0.84 (NS)	*ITGAM-ITGAX*
17	rs3803800	A	0.68	0.00	0.03	0.91 (NS)	*TNFSF13*
22	rs2412971	G	0.36	0.01	0.03	0.85 (NS)	*HORMAD2, LIF, OSM*
