# Supplementary material for: GWAS for serum galactose-deficient IgA1 implicates critical genes of the O-glycosylation pathway
Source: PLoS Genet. 2017 Feb 10;13(2):e1006609. doi: 10.1371/journal.pgen.1006609 (PMC5328405; doi:10.1371/journal.pgen.1006609)
Supplement: S2 Table — Serum Gd-IgA1 levels before and after adjustment for serum total IgA levels. (PDF) [file pgen.1006609.s007.pdf]

Supplementary Table 2. Combined association results for the 50 loci selected for replication: serum Gd-IgA1 levels before and after adjustment for serum total IgA levels.

					Serum Gd-IgA1 level										Serum Gd-IgA1 adjusted for total IgA										
					Discovery			Replication			All Cohorts				Discovery			Replication			All Cohorts				
Chr	Position (BP)	SNP	Test Allele	Other Allele	Effect	SE	P-value	Effect	SE	P-value	Effect	SE	P-value	Het P	Effect	SE	P-value	Effect	SE	P-value	Effect	SE	P-value	Het P	Genes in Locus
1	50326960	**rs6673246**	a	g	0.22	0.06	4.1E-04	-0.08	0.07	2.6E-01	0.09	0.05	5.1E-02	0.0003	0.02	0.04	6.4E-01	-0.05	0.05	2.6E-01	-0.01	0.03	6.8E-01	0.01	*ELAVL4,AGBL4*
1	77970910	**rs12030080**	a	g	0.2	0.05	1.3E-04	0.03	0.07	6.9E-01	0.13	0.04	1.1E-03	0.07	0.11	0.04	2.2E-03	0.02	0.04	6.3E-01	0.07	0.03	7.4E-03	0.34	*ZZZ3,USP33,NEXN,FAM73A*
1	95235559	**rs7533303**	t	g	-0.17	0.05	4.7E-04	-0.02	0.06	7.2E-01	-0.11	0.04	3.5E-03	0.05	-0.12	0.04	3.8E-04	-0.03	0.04	4.3E-01	-0.08	0.03	1.4E-03	0.16	*TMEM56,SLC44A3,CNN3,ALG14*
1	159944878	**rs12135523**	g	a	-0.17	0.05	2.9E-04	-0.04	0.06	4.6E-01	-0.12	0.04	1.0E-03	0.26	-0.11	0.03	7.0E-04	0.02	0.04	5.4E-01	-0.06	0.03	2.9E-02	0.05	*FCRLA,FCRLB,FCGR2B,FCGR3B*
1	163223590	**rs4656422**	a	g	0.26	0.07	2.5E-04	-0.08	0.08	3.1E-01	0.10	0.05	4.4E-02	0.04	0.24	0.05	3.7E-06	-0.05	0.06	4.1E-01	0.11	0.04	4.9E-03	0.01	*PBX1,LMX1*
1	244541684	**rs12048180**	a	g	0.17	0.05	4.1E-04	0.02	0.06	7.7E-01	0.10	0.04	3.8E-03	0.17	0.05	0.03	1.5E-01	0.02	0.04	6.0E-01	0.04	0.02	1.5E-01	0.48	*SMYD3*
2	180700025	**rs1401108**	t	c	-0.26	0.06	2.2E-05	0.00	0.08	9.8E-01	-0.16	0.05	8.8E-04	0.18	-0.13	0.05	5.9E-03	-0.03	0.06	5.8E-01	-0.09	0.04	1.3E-02	0.26	*CWC22*
3	131712006	**rs1497327**	c	t	-0.2	0.04	3.0E-06	-0.01	0.05	9.1E-01	-0.12	0.03	4.9E-04	0.002	-0.11	0.03	2.2E-04	0.001	0.03	9.9E-01	-0.06	0.02	6.0E-03	0.03	*COL29A1*
3	131713483	**rs2221010**	t	c	-0.21	0.04	1.9E-06	-0.02	0.05	7.4E-01	-0.13	0.03	1.4E-04	0.003	-0.11	0.03	2.7E-04	-0.0004	0.03	9.9E-01	-0.06	0.02	7.2E-03	0.02	*COL29A1*
4	169819026	**rs10452272**	t	c	-0.2	0.04	7.3E-06	-0.01	0.06	7.9E-01	-0.13	0.03	2.4E-04	0.15	-0.1	0.03	1.8E-03	-0.02	0.04	6.4E-01	-0.06	0.02	7.7E-03	0.30	*PALLD*
4	184547551	**rs9784568**	a	g	0.17	0.04	4.2E-05	0.02	0.05	7.4E-01	0.11	0.03	6.5E-04	0.10	0.05	0.03	1.0E-01	0.01	0.03	6.7E-01	0.03	0.02	1.3E-01	0.80	*WWC2,CLDN22,CDKN2AIP*
5	5127825	**rs270636**	a	g	0.23	0.06	3.9E-05	0.01	0.07	9.0E-01	0.14	0.04	1.4E-03	0.10	0.13	0.04	1.7E-03	-0.04	0.05	3.8E-01	0.05	0.03	8.0E-02	0.03	*ADAMTS16*
5	66384078	**rs462483**	g	a	-0.27	0.06	1.3E-05	0.08	0.08	2.9E-01	-0.13	0.05	6.9E-03	0.02	-0.18	0.04	5.4E-05	0.10	0.05	6.4E-02	-0.06	0.03	6.2E-02	0.003	*MAST4*
5	95307847	**rs2546199**	c	t	0.16	0.04	2.4E-04	-0.07	0.05	1.9E-01	0.12	0.03	2.4E-04	0.0004	0.09	0.03	3.2E-03	-0.06	0.04	8.9E-02	0.08	0.02	8.1E-04	0.24	*GLRX,ELL2*
5	95431099	**rs4538631**	t	c	0.17	0.04	3.5E-05	0.03	0.04	4.0E-01	0.10	0.03	4.5E-04	0.01	0.09	0.03	2.9E-03	0.03	0.03	3.9E-01	0.06	0.02	6.5E-03	0.35	*ELL2*
6	137842904	**rs9389508**	t	c	0.2	0.06	4.0E-04	-0.08	0.07	2.4E-01	0.09	0.04	5.1E-02	0.01	0.13	0.04	1.6E-03	-0.03	0.05	5.5E-01	0.06	0.03	5.0E-02	0.04	*OLIG3*
7	6400457	**rs836475**	a	g	-0.43	0.1	2.3E-05	0.14	0.14	3.4E-01	-0.24	0.08	3.7E-03	0.004	-0.25	0.07	3.1E-04	0.07	0.09	4.7E-01	-0.14	0.06	1.3E-02	0.01	*RAC1,MGC12966,KDELR2,DAGLB*
7	7213371	**rs13226913**	t	c	0.21	0.07	2.7E-03	0.21	0.05	8.9E-05	0.21	0.04	8.1E-07	0.73	0.2	0.05	2.2E-04	0.23	0.04	3.0E-08	0.22	0.03	3.2E-11	0.43	*C1GALT1*
7	7239965	**rs1008897**	g	a	0.21	0.07	2.6E-03	0.26	0.06	5.2E-06	0.24	0.04	5.4E-08	0.94	0.19	0.06	4.6E-04	0.22	0.04	4.6E-07	0.21	0.03	9.1E-10	0.93	*C1GALT1*
7	43345369	**rs978056**	a	g	-0.17	0.04	5.1E-05	-0.09	0.04	1.3E-02	-0.13	0.03	5.6E-06	0.27	-0.1	0.03	1.2E-03	-0.07	0.03	7.5E-03	-0.08	0.02	3.3E-05	0.15	*HECW1*
7	73524506	**rs37624**	c	t	-0.14	0.05	1.6E-03	0.02	0.05	6.7E-01	-0.09	0.03	7.3E-03	0.13	-0.14	0.03	9.9E-06	0.05	0.04	2.2E-01	-0.10	0.02	3.6E-05	0.06	*GTF2IRD1,CLIP2*
7	157963900	**rs1670339**	t	c	0.22	0.05	5.0E-06	-0.01	0.06	9.3E-01	0.13	0.04	4.4E-04	0.08	0.14	0.03	2.0E-05	0.02	0.04	6.4E-01	0.09	0.03	3.7E-04	0.04	*PTPRN2*
8	34820072	**rs16883114**	g	a	-0.19	0.05	5.5E-05	0.07	0.05	2.1E-01	-0.08	0.04	2.8E-02	0.02	-0.07	0.03	3.2E-02	-0.001	0.03	9.7E-01	-0.04	0.02	1.1E-01	0.81	*non-genic*
9	1459317	**rs12002949**	a	g	-0.35	0.1	2.9E-04	-0.13	0.09	1.8E-01	-0.24	0.07	5.1E-04	0.26	-0.12	0.07	1.0E-01	-0.09	0.06	1.5E-01	-0.10	0.05	3.1E-02	0.87	*non-genic*
9	34545481	**rs12552724**	t	c	-0.23	0.09	8.0E-03	-0.08	0.11	4.8E-01	-0.18	0.07	1.2E-02	0.26	-0.24	0.07	3.4E-04	-0.10	0.08	2.3E-01	-0.18	0.05	3.9E-04	0.09	*IL11RA,GALT,DNAI1,DCTN3,CNTFR*
9	129736531	**rs4836593**	a	g	-0.19	0.05	9.9E-05	-0.05	0.05	3.2E-01	-0.13	0.04	3.3E-04	0.13	-0.09	0.03	5.9E-03	-0.06	0.04	1.0E-01	-0.08	0.02	1.7E-03	0.60	*ST6GALNAC4,ST6GALNAC6*
10	3757522	**rs12570343**	g	a	-0.19	0.05	2.3E-04	-0.02	0.07	7.3E-01	-0.13	0.04	1.9E-03	0.14	-0.17	0.04	7.0E-06	0.04	0.04	3.3E-01	-0.08	0.03	5.1E-03	0.002	*KLF6*
10	5944924	**rs2203197**	t	c	0.23	0.06	7.5E-05	-0.08	0.07	2.3E-01	0.10	0.04	2.0E-02	0.02	0.1	0.04	1.4E-02	-0.05	0.05	3.0E-01	0.04	0.03	2.3E-01	0.12	*FBXO18,ANKRD16*
10	7183436	**rs10437543**	a	g	0.28	0.08	4.7E-04	-0.14	0.10	1.7E-01	0.12	0.06	5.5E-02	0.05	0.14	0.06	1.4E-02	-0.13	0.07	5.4E-02	0.03	0.04	5.0E-01	0.07	*SFMBT2*
10	12854934	**rs2493775**	g	a	-0.15	0.04	6.3E-04	0.03	0.05	5.8E-01	-0.10	0.03	3.2E-03	0.0002	-0.07	0.03	3.2E-02	0.001	0.03	9.8E-01	-0.04	0.02	1.1E-01	0.02	*CAMK1D*
10	18673783	**rs7919793**	g	a	-0.22	0.06	3.9E-04	0.00	0.08	9.9E-01	-0.14	0.05	5.4E-03	0.02	-0.08	0.05	9.0E-02	0.05	0.06	4.0E-01	-0.03	0.04	4.5E-01	0.21	*CACNB2*
10	18713106	**rs17611696**	t	c	-0.26	0.07	3.6E-04	-0.06	0.06	3.5E-01	-0.14	0.05	2.8E-03	0.070	-0.05	0.05	3.2E-01	-0.01	0.05	8.7E-01	-0.03	0.04	4.5E-01	0.42	*CACNB2*
10	29515875	**rs2532747**	a	g	0.19	0.04	1.0E-05	-0.01	0.05	8.4E-01	0.11	0.03	1.0E-03	0.01	0.1	0.03	1.1E-03	0.01	0.04	7.9E-01	0.06	0.02	8.1E-03	0.14	*non-genic*
10	117426242	**rs2804144**	a	g	-0.21	0.05	4.9E-05	0.03	0.06	6.6E-01	-0.11	0.04	4.6E-03	0.02	-0.11	0.04	2.5E-03	0.07	0.04	7.7E-02	-0.03	0.03	2.8E-01	0.02	*ATRNL1*
11	12873618	**rs7941340**	c	t	0.2	0.05	9.0E-05	0.03	0.06	5.5E-01	0.13	0.04	9.4E-04	0.15	0.12	0.04	1.3E-03	0.06	0.04	1.2E-01	0.09	0.03	6.8E-04	0.68	*TEAD1*
12	3001940	**rs7300221**	c	t	-0.34	0.09	8.7E-05	-0.07	0.09	4.6E-01	-0.21	0.06	7.5E-04	0.08	-0.15	0.07	3.0E-02	-0.08	0.07	2.3E-01	-0.12	0.05	1.7E-02	0.07	*TEAD4*
12	72715377	**rs1829127**	g	a	0.19	0.05	5.1E-05	-0.06	0.05	2.7E-01	0.08	0.04	2.1E-02	0.01	0.11	0.03	1.7E-03	-0.01	0.04	8.0E-01	0.05	0.03	3.2E-02	0.15	*non-genic*
13	74102084	**rs1359689**	t	c	-0.25	0.08	2.6E-03	0.05	0.09	5.6E-01	-0.12	0.06	6.0E-02	0.13	-0.27	0.07	3.2E-05	0.15	0.07	3.8E-02	-0.08	0.05	1.0E-01	0.0003	*KLF12*
13	94633634	**rs1887162**	t	g	-0.22	0.05	3.2E-05	0.08	0.06	2.0E-01	-0.09	0.04	2.7E-02	0.003	-0.15	0.04	5.5E-05	0.04	0.04	3.6E-01	-0.06	0.03	1.8E-02	0.02	*ABCC4*
15	77945097	**rs7177659**	c	a	-0.14	0.04	6.2E-04	0.08	0.05	1.3E-01	-0.05	0.03	9.3E-02	0.01	-0.11	0.03	3.5E-04	0.00	0.03	9.1E-01	-0.06	0.02	5.5E-03	0.13	*ST20,MTHFS,C15orf37,BCL2A1*
15	81872699	**rs8042077**	t	g	-0.2	0.04	1.0E-06	-0.01	0.04	7.7E-01	-0.10	0.03	3.7E-04	0.02	-0.12	0.03	4.0E-05	-0.01	0.03	8.1E-01	-0.06	0.02	2.7E-03	0.04	*SH3GL3*
16	8088809	**rs11865277**	t	c	0.44	0.1	5.0E-06	0.10	0.12	4.3E-01	0.30	0.07	4.8E-05	0.12	0.34	0.08	5.1E-05	-0.01	0.09	9.5E-01	0.18	0.06	3.4E-03	0.01	*non-genic*
16	23797562	**rs8063132**	g	a	-0.34	0.08	5.6E-05	0.00	0.06	9.8E-01	-0.12	0.05	1.8E-02	0.01	-0.21	0.07	2.5E-03	0.06	0.05	2.1E-01	-0.03	0.04	4.8E-01	0.06	*PRKCB1*
18	23086021	**rs16943399**	a	g	-0.2	0.06	4.9E-04	0.06	0.07	3.5E-01	-0.09	0.04	3.5E-02	0.05	-0.04	0.04	2.9E-01	0.002	0.04	9.6E-01	-0.02	0.03	4.4E-01	0.05	*non-genic*
19	39052469	**rs11882105**	g	a	0.18	0.04	3.3E-05	0.03	0.05	5.2E-01	0.12	0.03	3.5E-04	0.16	0.07	0.03	2.0E-02	-0.04	0.03	2.8E-01	0.02	0.02	3.2E-01	0.22	*KCTD15,CHST8*
19	56063231	**rs1506684**	t	c	-0.18	0.05	7.9E-05	-0.03	0.05	6.3E-01	-0.12	0.04	9.0E-04	0.17	-0.08	0.03	1.4E-02	-0.03	0.04	4.9E-01	-0.06	0.03	2.1E-02	0.48	*KLK3*
20	8499344	**rs2103653**	t	c	0.22	0.05	4.6E-05	0.06	0.06	3.1E-01	0.15	0.04	1.8E-04	0.32	0.12	0.04	2.2E-03	0.004	0.04	9.2E-01	0.07	0.03	1.9E-02	0.14	*PLCB1*
22	28200951	**rs5752924**	g	a	0.23	0.06	1.8E-04	0.00	0.07	9.5E-01	0.14	0.05	3.4E-03	0.02	0.07	0.04	9.7E-02	0.01	0.05	8.2E-01	0.04	0.03	1.6E-01	0.11	*RFPL1,NEFH*
22	28402759	**rs737787**	c	t	0.26	0.06	4.1E-05	0.09	0.08	2.3E-01	0.19	0.05	9.0E-05	0.26	0.07	0.05	1.4E-01	0.01	0.05	8.5E-01	0.04	0.03	2.2E-01	0.05	*ZMAT5,UCRC,THOC5,NIPSNAP1,NF2*
X	119642838	**rs5910940**	c	t	-0.15	0.05	1.6E-03	-0.12	0.04	8.5E-04	-0.13	0.03	5.0E-06	0.69	-0.13	0.03	2.2E-04	-0.11	0.03	3.8E-05	-0.14	0.03	2.7E-08	0.88	*C1GALT1C1*
X	119698292	**rs2196262**	a	g	0.18	0.05	1.3E-04	0.10	0.04	7.7E-03	0.13	0.03	9.2E-06	0.11	0.11	0.03	1.2E-03	0.10	0.03	3.3E-04	-0.12	0.02	1.4E-06	0.46	*C1GALT1C1*
