# Supplementary material for: GWAS for serum galactose-deficient IgA1 implicates critical genes of the O-glycosylation pathway
Source: PLoS Genet. 2017 Feb 10;13(2):e1006609. doi: 10.1371/journal.pgen.1006609 (PMC5328405; doi:10.1371/journal.pgen.1006609)
Supplement: S3 Table — The power was estimated for a range of effect sizes expressed as fraction of total variance of the quantitative trait explained by a genetic variant (columns). The assumptions include: standard normal trait distribution, additive risk model, no heterogeneity, marker allelic frequency of 0.25, perfect LD between a marker and a causal allele, a follow-up significance threshold of P<5×10^−4^ (top row) and a joint significance level of P<5×10^−8^ (bottom row). Shaded in red is the study detection limit corresponding to alleles explaining 1.5% of total variance. (PDF) [file pgen.1006609.s008.pdf]

Supplementary Table 3. Study power.

The power was estimated for a range of effect sizes expressed as fraction of total variance of the quantitative trait explained by a genetic variant (columns). The assumptions include: standard normal trait distribution, additive risk model, no heterogeneity, marker allelic frequency of 0.25, perfect LD between a marker and a causal allele, a follow-up significance threshold of $P<5\times10^{-4}$ (top row) and a joint significance level of $P<5\times10^{-8}$ (bottom row). Shaded in red is the study detection limit corresponding to alleles explaining 1.5% of total variance.

	**Variance Explained**						
	**1.0%**	**1.5%**	**2.0%**	**2.5%**	**3.0%**	**3.5%**	**4.0%**
**Discovery Cohorts** (N=1,195, alpha = $5x10^{-4}$)	49%	78%	92%	98%	99%	100%	100%
**All Cohorts Combined** (N=2,633, alpha = $5x10^{-8}$)	38%	80%	97%	99%	100%	100%	100%
