# Supplementary material for: GWAS for serum galactose-deficient IgA1 implicates critical genes of the O-glycosylation pathway
Source: PLoS Genet. 2017 Feb 10;13(2):e1006609. doi: 10.1371/journal.pgen.1006609 (PMC5328405; doi:10.1371/journal.pgen.1006609)
Supplement: S4 Table — The fraction of total variance explained was estimated by regressing individual genetic predictors (additive coding) against the outcome of standardized residuals for the trait (Gd-IgA1 levels adjusted for age, case-control status, and serum total IgA levels) and deriving R2 for the regression model. The total variance explained across multiple cohorts was calculated as an average fraction of explained variance for individual cohorts weighted by cohort size. The variance explained by the C1GALT1 locus was calculated by including both rs13226913 and rs1008897 in the regression model. For C1GALT1C1 locus, both rs5910940 and rs2196262 were included under additive coding. The total variance explained jointly by C1GALT1 and C1GALT1C1 loci was calculated by including all four SNP predictors from these loci in a single regression model. (PDF) [file pgen.1006609.s009.pdf]

Supplementary Table 4. Total variance explained by genome-wide significant loci.

The fraction of total variance explained was estimated by regressing individual genetic predictors (additive coding) against the outcome of standardized residuals for the trait (Gd-IgA1 levels adjusted for age, case-control status, and serum total IgA levels) and deriving R2 for the regression model. The total variance explained across multiple cohorts was calculated as an average fraction of explained variance for individual cohorts weighted by cohort size. The variance explained by the *C1GALT1* locus was calculated by including both rs13226913 and rs1008897 in the regression model. For *C1GALT1C1* locus, both rs5910940 and rs2196262 were included under additive coding. The total variance explained jointly by *C1GALT1* and *C1GALT1C1* loci was calculated by including all four SNP predictors from these loci in a single regression model.

Cohort(s)	N	*C1GALT1 locus*	*C1GALT1C1 locus*	*C1GALT1+C1GALT1C1 loci*
**Asian Discovery**	950	0.8%	1.2%	2.1%
**Asian Replication**	653	1.0%	1.1%	2.0%
**All Asians**	**1,603**	**0.9%**	**1.2%**	**2.0%**
**European Discovery**	245	3.6%	1.2%	5.0%
**European Replication**	785	4.3%	3.3%	7.8%
**All Europeans**	**1,030**	**4.2%**	**2.8%**	**7.1%**
**All Cohorts**	2,633	2.2%	1.8%	4.0%
