# Supplementary material for: GWAS for serum galactose-deficient IgA1 implicates critical genes of the O-glycosylation pathway
Source: PLoS Genet. 2017 Feb 10;13(2):e1006609. doi: 10.1371/journal.pgen.1006609 (PMC5328405; doi:10.1371/journal.pgen.1006609)
Supplement: S5 Table — Each SNP that reached genome-wide significance in our study was conditioned on all other SNPs that reached genome-wide significance, one at a time. Highlighted in red are independent effects for markers located within the same locus after conditioning on the other significant marker within the same locus. Notably, conditioning within each locus demonstrates residual effects, while mutual conditioning across loci strengthens the association signal at each locus. Because chromosome X markers are included in these analyses, all models were sub-stratified based on sex; the conditioning was first performed within each sub-cohort, then the results were combined using fixed effects meta-analysis. In all analyses, markers were coded under an additive model and the Gd-IgA1-increasing allele was used as a test allele. StdErr. Standard error. (PDF) [file pgen.1006609.s010.pdf]

Supplementary Table 5. Mutual conditioning across the genome-wide significant loci

Each SNP that reached genome-wide significance in our study was conditioned on all other SNPs that reached genome-wide significance, one at a time. Highlighted in red are independent effects for markers located within the same locus after conditioning on the other significant marker within the same locus. Notably, conditioning within each locus demonstrates residual effects, while mutual conditioning across loci strengthens the association signal at each locus. Because chromosome X markers are included in these analyses, all models were sub-stratified based on sex; the conditioning was first performed within each sub-cohort, then the results were combined using fixed effects meta-analysis. In all analyses, markers were coded under an additive model and the Gd-IgA1-increasing allele was used as a test allele. StdErr. Standard error.

		Conditional Analysis			
**Locus**	**Test SNP**	**Conditioning SNP**	**Effect**	**StdErr**	**P-value**
C1GALT1	rs13226913	none	0.21	0.03	1.1E-12
	rs13226913	rs1008897	0.16	0.04	3.9E-05
	rs13226913	rs5910940	0.22	0.03	3.4E-13
	rs13226913	rs2196262	0.22	0.03	7.2E-13
	rs1008897	none	0.20	0.03	4.0E-10
	rs1008897	rs13226913	0.11	0.04	1.0E-02
	rs1008897	rs5910940	0.20	0.03	1.6E-10
	rs1008897	rs2196262	0.20	0.03	1.3E-10
C1GALT1C1	rs5910940	none	0.14	0.03	2.7E-08
	rs5910940	rs13226913	0.14	0.03	1.4E-08
	rs5910940	rs1008897	0.14	0.03	3.4E-08
	rs5910940	rs2196262	0.11	0.03	1.0E-04
	rs2196262	none	0.12	0.02	1.4E-06
	rs2196262	rs13226913	0.12	0.02	4.4E-07
	rs2196262	rs1008897	0.12	0.02	6.8E-07
	rs2196262	rs5910940	0.08	0.03	2.3E-03
