# Supplementary material for: GWAS for serum galactose-deficient IgA1 implicates critical genes of the O-glycosylation pathway
Source: PLoS Genet. 2017 Feb 10;13(2):e1006609. doi: 10.1371/journal.pgen.1006609 (PMC5328405; doi:10.1371/journal.pgen.1006609)
Supplement: S7 Table — (PDF) [file pgen.1006609.s012.pdf]

Supplementary Table 7. Expression QTL effects of rs13226913 across multiple tissue types.

Database	eSNP	Test Allele	Alt. Allele	Test Allele Effect (+/-)	Gene	P-Value	Tissue
Blood eQTL	rs13226913	**T**	C	-	*C1GALT1*	3.86E-23	Whole Blood
GETx	rs13226913	**T**	C	-	*C1GALT1*	1.30E-25	Nerve - Tibial
GETx	rs13226913	**T**	C	-	*C1GALT1*	3.40E-24	Thyroid
GETx	rs13226913	**T**	C	-	*C1GALT1*	6.50E-18	Testis
GETx	rs13226913	**T**	C	-	*C1GALT1*	2.00E-16	Adipose - Subcutaneous
GETx	rs13226913	**T**	C	-	*C1GALT1*	2.70E-15	Esophagus - Muscularis
GETx	rs13226913	**T**	C	-	*C1GALT1*	2.30E-14	Pancreas
GETx	rs13226913	**T**	C	-	*C1GALT1*	3.60E-13	Artery - Tibial
GETx	rs13226913	**T**	C	-	*C1GALT1*	2.60E-12	Skin - Sun Exposed (Lower leg)
GETx	rs13226913	**T**	C	-	*C1GALT1*	4.30E-10	Stomach
GETx	rs13226913	**T**	C	-	*C1GALT1*	4.40E-10	Lung
GETx	rs13226913	**T**	C	-	*C1GALT1*	1.20E-09	Adrenal Gland
GETx	rs13226913	**T**	C	-	*C1GALT1*	6.50E-09	Esophagus - Gastroesophageal Junction
GETx	rs13226913	**T**	C	-	*C1GALT1*	1.10E-08	Prostate
GETx	rs13226913	**T**	C	-	*C1GALT1*	1.10E-08	Colon - Transverse
GETx	rs13226913	**T**	C	-	*C1GALT1*	1.30E-08	Muscle - Skeletal
GETx	rs13226913	**T**	C	-	*C1GALT1*	2.50E-08	Adipose - Visceral (Omentum)
GETx	rs13226913	**T**	C	-	*C1GALT1*	2.80E-08	Brain - Cerebellum
GETx	rs13226913	**T**	C	-	*C1GALT1*	6.40E-08	Pituitary
GETx	rs13226913	**T**	C	-	*C1GALT1*	7.10E-08	Brain - Hippocampus
GETx	rs13226913	**T**	C	-	*C1GALT1*	7.90E-08	Brain - Nucleus accumbens (basal ganglia)
GETx	rs13226913	**T**	C	-	*C1GALT1*	1.10E-07	Heart - Atrial Appendage
GETx	rs13226913	**T**	C	-	*C1GALT1*	1.40E-07	Esophagus - Mucosa
GETx	rs13226913	**T**	C	-	*C1GALT1*	2.40E-07	Spleen
GETx	rs13226913	**T**	C	-	*C1GALT1*	2.50E-07	Brain - Cerebellar Hemisphere
GETx	rs13226913	**T**	C	-	*C1GALT1*	3.40E-07	Brain - Putamen (basal ganglia)
GETx	rs13226913	**T**	C	-	*C1GALT1*	4.50E-07	Brain - Cortex
GETx	rs13226913	**T**	C	-	*C1GALT1*	5.90E-07	Brain - Frontal Cortex (BA9)
GETx	rs13226913	**T**	C	-	*C1GALT1*	2.30E-06	Heart - Left Ventricle
GETx	rs13226913	**T**	C	-	*C1GALT1*	4.20E-06	Cells - Transformed fibroblasts

(+) increasing effect (-) decreasing effect
