# Supplementary material for: GWAS for serum galactose-deficient IgA1 implicates critical genes of the O-glycosylation pathway
Source: PLoS Genet. 2017 Feb 10;13(2):e1006609. doi: 10.1371/journal.pgen.1006609 (PMC5328405; doi:10.1371/journal.pgen.1006609)
Supplement: S8 Table — We explored two alternative genetic models (dominant and recessive) and compared these models using Bayesian Information Criterion (BIC). The best model is highlighted in red. While this analysis suggests an additive model for 4 out of 5 top markers, the effect of rs5910940 (C1GALT1C1 locus) is best explained by a T-allele dominant model. All analyses were stratified based on sex, explaining slight differences in effect estimates and p-values compared to Table 2. StdErr: standard error. (PDF) [file pgen.1006609.s013.pdf]

Supplementary Table 8. Exploration of alternative genetic models.

We explored two alternative genetic models (dominant and recessive) and compared these models using Bayesian Information Criterion (BIC). The best model is highlighted in red. While this analysis suggests an additive model for 4 out of 5 top markers, the effect of rs5910940 (*C1GALT1C1* locus) is best explained by a T-allele dominant model. All analyses were stratified based on sex, explaining slight differences in effect estimates and p-values compared to Table 2. StdErr: standard error.

		ADDITIVE MODEL*				DOMINANT MODEL**				RECESSIVE MODEL***			
**SNP**	**Allele**	**Effect**	**StdErr**	**P-value**	**BIC ^**	**Effect**	**StdErr**	**P-value**	**BIC ^**	**Effect**	**StdErr**	**P-value**	**BIC ^**
rs13226913	**T**	0.21	0.03	1.1E-12	6211.3	0.33	0.07	4.3E-07	6228.2	0.24	0.04	7.8E-10	6226.1
rs1008897	**G**	0.20	0.03	4.0E-10	6217.1	0.33	0.08	3.2E-05	6224.9	0.22	0.04	2.1E-08	6235.2
rs5910940	**T**	0.14	0.03	2.7E-08	6234.2	0.19	0.03	7.9E-09	6229.7	0.14	0.03	7.2E-06	6242.8
rs2196262	**A**	0.12	0.02	1.4E-06	6223.3	0.15	0.03	2.5E-06	6226.2	0.13	0.03	2.8E-05	6226.4
rs978056	**G**	0.08	0.02	6.7E-05	6199.9	0.11	0.04	2.1E-03	6206.3	0.11	0.03	6.0E-04	6203.6

* Additive model allele coding (0/1/2), sex- and cohort-stratified meta-analysis of all cohorts

** Recessive model allele coding (0/0/2), sex- and cohort-stratified meta-analysis of all cohorts

*** Dominant model allele coding (0/2/2), sex- and cohort-stratified meta-analysis of all cohorts

^ BIC was calculated for each model for cohort-, affection-, and sex-stratified analysis of all cohorts combined
