# Supplementary material for: GWAS for serum galactose-deficient IgA1 implicates critical genes of the O-glycosylation pathway
Source: PLoS Genet. 2017 Feb 10;13(2):e1006609. doi: 10.1371/journal.pgen.1006609 (PMC5328405; doi:10.1371/journal.pgen.1006609)
Supplement: S10 Table — The East Asians include the Chinese Discovery, the Chinese Replication, and the Japanese Replication cohorts (N = 1,603). The Europeans include the US discovery cohort (100% self-identified Whites), German, French, and US Replication cohorts (N = 1,030). The results for all ethnicity-defined cohorts were combined using fixed effects meta-analysis. Allelic frequencies were averaged within the ethnicity-defined cohorts. (PDF) [file pgen.1006609.s015.pdf]

Supplementary Table 10. Ethnicity-specific association results for the significant and suggestive loci.

The East Asians include the Chinese Discovery, the Chinese Replication, and the Japanese Replication cohorts (N=1,603). The Europeans include the US discovery cohort (100% self-identified Whites), German, French, and US Replication cohorts (N=1,030). The results for all ethnicity-defined cohorts were combined using fixed effects meta-analysis. Allelic frequencies were averaged within the ethnicity-defined cohorts.

				East Asians N=1,603					Europeans N=1,030					
**CHR**	**Position (BP)**	**SNP**	**Test Allele**	**Asian Freq.**	**Effect**	**SE**	**P-value**	**Hetero P-value**	**European Freq.**	**Effect**	**SE**	**P-value**	**Hetero P-value**	**Genes in Locus**
7	7213371	rs13226913	**T**	0.10	0.12	0.06	5.3E-02	0.41	0.47	0.26	0.04	2.7E-11	0.82	*C1GALT1*
7	7239965	rs1008897	**G**	0.07	0.17	0.06	5.0E-03	0.71	0.27	0.23	0.04	4.0E-08	0.87	*C1GALT1*
23	119642838	rs5910940	**T**	0.53	0.11	0.03	5.9E-05	0.43	0.54	0.13	0.03	1.4E-04	0.75	*C1GALT1C1*
23	119698292	rs2196262	**A**	0.48	0.09	0.03	9.0E-04	0.22	0.52	0.12	0.03	3.7E-04	0.24	*C1GALT1C1*
7	43345369	rs978056	**G**	0.45	0.11	0.02	2.1E-06	0.40	0.47	0.01	0.04	7.4E-01	0.44	*HECW1*
