# Supplementary material for: GWAS for serum galactose-deficient IgA1 implicates critical genes of the O-glycosylation pathway
Source: PLoS Genet. 2017 Feb 10;13(2):e1006609. doi: 10.1371/journal.pgen.1006609 (PMC5328405; doi:10.1371/journal.pgen.1006609)
Supplement: S11 Table — Minimum sample sizes (cases + controls) required to detect associations of the newly detected Gd-IgA1 loci with the risk of IgAN in East Asian, European, and bi-ethnic GWAS assuming observed effect sizes, 50% case proportion, α = 5 x 10^−8^, and power (1-β) of 80%, 90% and 99.9%. The variance explained by each locus was derived as in S4 Table. The observed ORs of disease per standard deviation of endophenotype were calculated based on logistic regression with case/control status as an outcome and standardized residuals of Gd-IgA1 (after adjustment for age, sex, cohort, and total IgA levels) as a predictor. Separate estimates were obtained for our East Asian, European, and bi-ethnic cohorts. The calculations were performed within the framework of Mendelian Randomization, as previously proposed by Brion et al. Int J Epidemiol 42,1497–501 (2013) and implemented in the online calculator at https://cnsgenomics.shinyapps.io/mRnd/ (PDF) [file pgen.1006609.s016.pdf]

Supplementary Table 11. Sample sizes required for testing new Gd-IgA1 loci for association with IgA nephropathy.

Minimum sample sizes (cases + controls) required to detect associations of the newly detected Gd-IgA1 loci with the risk of IgAN in East Asian, European, and bi-ethnic GWAS assuming observed effect sizes, 50% case proportion, $\alpha = 5 \times 10^{-8}$, and power (1-β) of 80%, 90% and 99.9%. The variance explained by each locus was derived as in Supplementary Table 4. The observed ORs of disease per standard deviation of endophenotype were calculated based on logistic regression with case/control status as an outcome and standardized residuals of Gd-IgA1 (after adjustment for age, sex, cohort, and total IgA levels) as a predictor. Separate estimates were obtained for our East Asian, European, and bi-ethnic cohorts. The calculations were performed within the framework of Mendelian Randomization, as previously proposed by Brion et al. *Int J Epidemiol* 42,1497-501 (2013) and implemented in the online calculator at https://cnsgenomics.shinyapps.io/mRnd/

	Mendelian Randomization Instrument	Total Variance in Gd-IgA1 Explained by the Instrument	Observed OR of Disease per SD of Gd-IgA1 (95%CI)	Minimum Sample Size Required for 80% Power	Minimum Sample Size Required for 90% Power	Minimum Sample Size Required for 99.9% Power
**Biethnic**	*C1GALT1*	2.20%	1.53 (1.40-1.68)	39,218	44,893	72,252
	C1GALT1C1	1.80%	1.53 (1.40-1.68)	47,933	54,869	88,308
**European**	*C1GALT1*	4.20%	1.49 (1.31-1.72)	23,406	26,792	43,120
	C1GALT1C1	2.80%	1.49 (1.31-1.72)	35,108	40,188	64,680
**East Asian**	*C1GALT1*	0.90%	1.56 (1.37-1.78)	87,554	100,223	161,302
	C1GALT1C1	1.20%	1.56 (1.37-1.78)	65,665	75,167	120,977
